# Design, Synthesis, and In Vitro, In Silico and In Cellulo Evaluation of New Pyrimidine and Pyridine Amide and Carbamate Derivatives as Multi-Functional Cholinesterase Inhibitors

**DOI:** 10.3390/ph15060673

**Published:** 2022-05-27

**Authors:** Martina Bortolami, Fabiana Pandolfi, Valeria Tudino, Antonella Messore, Valentina Noemi Madia, Daniela De Vita, Roberto Di Santo, Roberta Costi, Isabella Romeo, Stefano Alcaro, Marisa Colone, Annarita Stringaro, Alba Espargaró, Raimon Sabatè, Luigi Scipione

**Affiliations:** 1 Department of Scienze di Base e Applicate per l’Ingegneria, Sapienza University of Rome, Via Castro Laurenziano 7, 00185 Rome, Italy; martina.bortolami@uniroma1.it (M.B.); fabiana.pandolfi@uniroma1.it (F.P.); 2 Department of Chimica e Tecnologia del Farmaco, Sapienza University of Rome, Piazzale Aldo Moro 5, 00185 Rome, Italy; valeria.tudino@uniroma1.it (V.T.); antonella.messore@uniroma1.it (A.M.); valentinanoemi.madia@uniroma1.it (V.N.M.); roberto.disanto@uniroma1.it (R.D.S.); roberta.costi@uniroma1.it (R.C.); 3 Department of Environmental Biology, Sapienza University of Rome, Piazzale Aldo Moro 5, 00185 Rome, Italy; daniela.devita@uniroma1.it; 4 Instituto Pasteur, Fondazione Cenci Bolognetti, Sapienza University of Rome, Piazzale Aldo Moro 5, 00185 Rome, Italy; 5 Net4Science Academic Spin-Off, Università degli Studi “Magna Græcia” di Catanzaro, Campus “S. Venuta”, Viale Europa, 88100 Catanzaro, Italy; isabella.romeo@unicz.it; 6 Dipartimento di Scienze della Salute, Università degli Studi “Magna Græcia” di Catanzaro, 88100 Catanzaro, Italy; 7 National Center for Drug Research and Evaluation, Istituto Superiore di Sanità, 00161 Rome, Italy; marisa.colone@iss.it (M.C.); annarita.stringaro@iss.it (A.S.); 8 Department of Pharmacy and Pharmaceutical Technology and Physical-Chemistry, Faculty of Pharmacy and Food Sciences, University of Barcelona, 08007 Barcelona, Spain; aespargaro@ub.edu (A.E.); rsabate@ub.edu (R.S.); 9 Institute of Nanoscience and Nanotechnology (IN^2^UB), 08028 Barcelona, Spain

**Keywords:** acetylcholinesterase inhibitors, butyrylcholinesterase inhibitors, multifunctional compounds, amyloid aggregation, tau aggregation, metal chelation

## Abstract

Alzheimer disease is an age-linked neurodegenerative disorder representing one of the greatest medical care challenges of our century. Several drugs are useful in ameliorating the symptoms, even if none could stop or reverse disease progression. The standard approach is represented by the cholinesterase inhibitors (ChEIs) that restore the levels of acetylcholine (ACh) by inhibiting the acetylcholinesterase (AChE). Still, their limited efficacy has prompted researchers to develop new ChEIs that could also reduce the oxidative stress by exhibiting antioxidant properties and by chelating the main metals involved in the disease. Recently, we developed some derivatives constituted by a 2-amino-pyrimidine or a 2-amino-pyridine moiety connected to various aromatic groups by a flexible amino-alkyl linker as new dual inhibitors of AChE and butyrylcholinesterase (BChE). Following our previous studies, in this work we explored the role of the flexible linker by replacing the amino group with an amide or a carbamic group. The most potent compounds showed higher selectivity against BChE in respect to AChE, proving also to possess a weak anti-aggregating activity toward Aβ_42_ and tau and to be able to chelate Cu^2+^ and Fe^3+^ ions. Molecular docking and molecular dynamic studies proposed possible binding modes with the enzymes. It is noteworthy that these compounds were predicted as BBB-permeable and showed low cytotoxicity on the human brain cell line.

## 1. Introduction

Alzheimer’s disease (AD) is a neurodegenerative disorder with a chronic and progressive course, exhibited by memory loss and impaired cognitive abilities. As is well known, the major risk factor associated with the onset of dementia is age. The elderly population is constantly growing in the world and life expectancy is increasing at a steady rate [1]. The weight of this aging contributes to confirming the estimates of numerous international epidemiological studies that count 55 million cases of people with dementia in the world and predict, in 2030, a growth of more than 78 million, which could add, in the next twenty years, a figure exceeding 139 million people, with the vast majority concentrated in developing countries [2].

The pathogenesis of AD is complex and, since the currently available therapy brings limited benefits, finding new molecules that act on more specific targets is one of the most promising strategies in the search for effective treatments [3,4,5,6]. The disease is characterized by intracellular neurofibrillary tangles, due to the hyperphosphorylation and aggregation of tau protein, and by senile plaques, extracellular deposits of neurotoxic insoluble fibrils of β-amyloid peptide (Aβ) [7,8,9]. The latter is a metalloprotein with high binding affinity for biometals essential for cellular homeostasis, such as Cu^2+^, Zn^2+^ and Fe^3+^, and high standard redox potential that involves the reduction of these ions and the trapping of molecular oxygen, giving rise to radical species causing oxidation of proteins, DNA and RNA and increased lipid peroxidation [10].

These lesions severely impair cholinergic transmission, so the pharmacological approach is primarily based on restoring acetylcholine (ACh) levels through the use of cholinesterase inhibitors (ChEIs). In the enzymatic pocket of cholinesterase (ChEs) there are two binding sites: the catalytic active site (CAS) and the peripheral anionic site (PAS) [11,12,13]. The involvement of acetylcholinesterase (AChE), through PAS, in the fibrillar aggregation of Aβ has given new meaning to the therapeutic use of AChE inhibitors with mixed or non-competitive mechanisms, as they act both by restoring ACh levels and interfering with Aβ aggregation [14,15,16]. Several studies also reported the role of butyrylcholinesterase (BChE) in the deposition of Aβ plaques and in the compensation of AChE function in ongoing AD patients [17,18,19,20].

Other important factors in the complex etiology of AD are metal ions (mainly copper, zinc and iron), probably considered responsible for the protein misfolding, and the oxidative stress [21,22,23].

During the last few years, scientific advances have inspired researchers to study new pharmacological therapies focused more on the pathophysiological events of the AD [24]. In particular, one of the central choices of medicinal chemistry research is the development of multitarget or multifunctional ChEIs [25,26,27]. Some of these studies suggest that a possible way forward could be the development of new therapeutic agents with anticholinesterasic activity that also mitigate oxidative stress both directly, by exerting antioxidant functions, and indirectly, through the chelation of the main metal ions involved in the generation of reactive oxygen species [28,29]. 

In our previous works, we synthetized and studied new molecules, characterized by two small aromatic moieties separated by various functionalized linkers, designed as new dual binding AChE and BChE inhibitors, able to bind both CAS and PAS of the enzymes, as well as being endowed with other properties, such as anti-aggregating activity, chelating and antioxidant abilities, which are potentially interesting in the light of the multifunctional nature of AD [30,31,32]. From these molecules, we recently developed some derivatives constituted by a 2-amino-pyrimidine or a 2-amino-pyridine moiety connected to various aromatic groups by a flexible amino-alkyl linker [32]. In this way, we obtained mixed ChEs inhibitors, in which the two aromatic moieties and the amine group inserted into the linear alkyl chain are able to interact with the amino acids located in the CAS, PAS and mid-gorge cavities by means of π−π stacking and π−cation interactions, acting as dual binding site inhibitors, as highlighted by in silico analyses. Moreover, the 2-amino-pyrimidinic or 2-amino-pyridinic moieties confer chelating activity to the compounds, due to the presence of the two adjacent nitrogen atoms. Noteworthy, these molecules are also characterized by low toxicity toward human brain cells and good predicted physicochemical and ADME parameters.

**Figure 1:**
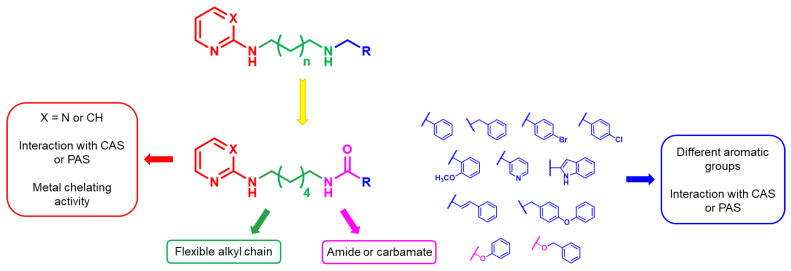
Rational design of the 2-amino-pyrimidine or 2-amino-pyridine amide or carbamate derivatives starting from diamine derivatives.

As a development of these works, we designed new pyrimidine and pyridine derivatives, replacing the amino group inserted into the alkyl chain with an amide or a carbamic group (Figure 1), and we studied these molecules in vitro, in silico and in cellulo to assay their ChEs inhibitory activity, chelating ability and anti-aggregating properties. The amide or carbamic groups were chosen to confer a major rigidity to the alkyl chain, as well as to form hydrogen bonds with the amino acids of the enzymes, with the aim to obtain a better and more stable orientation of the two aromatic moieties into the enzymatic gorge. Moreover, the carbamic group could establish covalent interaction with the serine of the catalytic site, forming a carbamoyl–cholinesterase complex like other ChEs carbamoylating inhibitors, contributing directly to the inhibitory action of the molecules. Based on the previous results, the length of the alkyl chain was chosen was six methylene units, to cover the distance between CAS and PAS. Among the aromatic groups introduced on one side of the aliphatic chain, besides the unsubstituted or substituted phenyl rings already studied for the amine derivatives of the previous work, bulkier groups, such as 4-phenoxybezyl or cinnamic one, were selected with the purpose to study the effects of an increase in the size of the molecules in the capacity to inhibit both ChEs or, selectively, BChE.

The synthesized compounds were screened against *Electrophorus electricus* AChE (*Ee*AChE) and equine BChE (*eq*BChE) by means of Ellman’s spectrophotometric method in order to evaluate their inhibition potency and selectivity. The studied compounds appear more selective towards *eq*BChE than *Ee*AChE, and the most potent *eq*BChE inhibitors were further studied to identify inhibition mechanism and inhibition constants. Molecular docking and molecular dynamic studies were carried out with the aim of identifying the possible interactions among these molecules and the amino acids located in the CAS, PAS and mid-gorge region of both enzymes and to explain the selectivity towards *eq*BChE. Further studies were conducted on the most active compounds with the aim to evaluate their Cu^2+^ and Fe^3+^ chelation ability by means of UV–Vis spectroscopic analyses, while in cellulo studies on *E. coli* cells were carried out to reveal their anti-aggregating activity toward Aβ_42_ and tau. Finally, for these compounds, the ability to permeate the BBB was predicted by in silico simulation and the cytotoxicity on a human brain cell line was also evaluated.

## 2. Results and Discussion

### 2.1. Chemistry

**Scheme 1:**
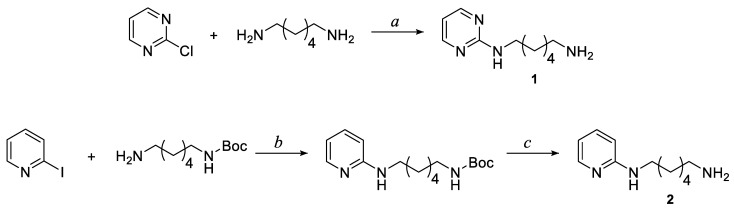
Synthesis of pyrimidine and pyridine intermediates **1** and **2**. *Reagents and conditions*: *a* TEA (1 eq), CH_3_OH, reflux, 18h; *b* CuI (0.05 eq), Cs_2_CO_3_ (2 eq), 2-isobutyrylcyclohexanone (0.2 eq), DMF, N_2_, 40 °C, 21 h; *c* TFA (20 eq), CH_2_Cl_2_, rt, 3.5 h.

The pyrimidine and pyridine intermediates **1** and **2**, used for the synthesis of corresponding amide and carbamate derivatives, were obtained as illustrated in Scheme 1 following the procedures previously reported [31].

**Scheme 2:**
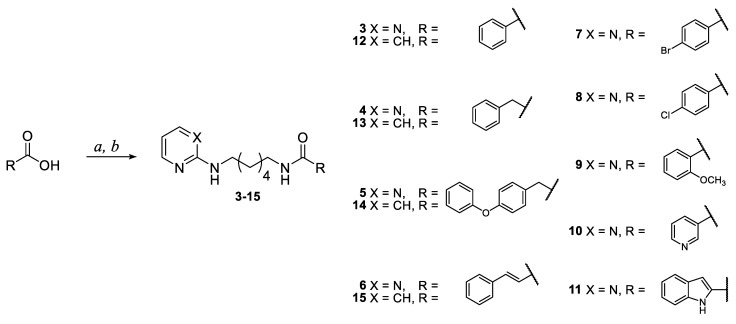
Synthesis of pyrimidine and pyridine amide derivatives **3–15**. *Reagents and conditions*: *a* CDI (1 eq), CH_3_CN, reflux, 5 h; *b N*^1^-(pyrimidin-2-yl)hexane-1,6-diamine (**1**) (for **3–11**) or *N*^1^-(pyridin-2-yl)hexane-1,6-diamine (**2**) (for **12–15**) (1 eq), reflux, 18 h.

The pyrimidine and pyridine amide compounds **3–15** were prepared as described in Scheme 2. Initially, the opportune carboxylic acid was activated using carbonyldiimidazole (CDI) in acetonitrile at reflux; the reaction was monitored through IR spectroscopy, evaluating the appearance of two carbonyl stretching bands around 1735 and 1700 cm^−1^, concerning the CDI-carboxylic acid intermediate. At this point, pyrimidine intermediate **1** or pyridine intermediate **2** was added and the reaction was refluxed overnight. The compounds were purified by column chromatography on silica gel and/or by crystallisation. The structures of final compounds were confirmed by spectroscopic analysis; as an example, in the ^1^H-NMR spectra of amide derivatives the shifts of the signals due to the -CH_2_-NH- groups were observed from 2.70 ppm (-CH_2_-NH_2_ in intermediates **1** and **2**) to 3.38–2.99 ppm (-CH_2_-NH-C=O in amide derivatives).

**Scheme 3:**
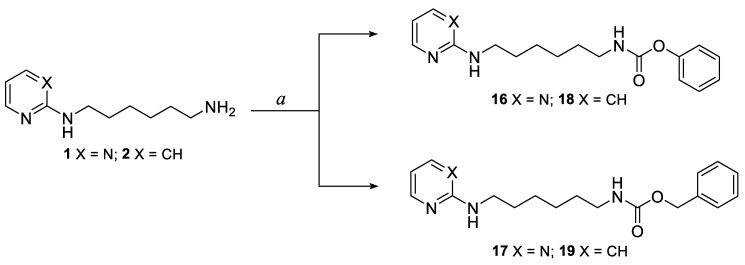
Synthesis of pyrimidine and pyridine carbamate derivatives **16–19**. *Reagents and conditions*: *a* TEA (1 eq), phenyl chloroformate (for **16**, **18**) or benzyl chloroformate (for **17**, **19**) (1 eq), CHCl_3_, room temperature, 3–18 h.

The pyrimidine and pyridine carbamate derivatives **16**–**19** were synthetized following the procedure described in Scheme 3. Pyrimidine intermediate **1** or pyridine intermediate **2** was dissolved in chloroform and then triethylamine and the opportune chloroformate were added. The reactions were monitored by ESI-MS; although the formation of the desired compounds was detected already after 3 h of reaction, the best yields were obtained leaving the mixture under stirring overnight (e.g., compound **19**: yield 33% after stirring for 18 h, 15% after stirring for 3 h). The compounds were purified by column chromatography on silica gel. The structures of final compounds were confirmed by spectroscopic analysis; as an example, in the ^1^H-NMR spectra of carbamate derivatives the shifts of the signals due to the -CH_2_-NH- groups were observed from 2.70 ppm (-CH_2_-NH_2_ in intermediates **1** and **2**) to 3.18–2.97 ppm (-CH_2_-NH-C=O in carbamates derivatives).

The detailed synthetic procedures, the analytical and spectroscopic data of the synthesized compounds, are reported in the experimental section and agree with the proposed structures.

### 2.2. Enzymatic Assays

Regarding the synthesized compounds enzymatic inhibition studies were carried out on *Electrophorus electricus* AChE (*Ee*AChE) and equine BChE (*eq*BChE), according to Ellman’s spectrophotometric method [33]. Initially, for each compound, except those insoluble under experimental conditions (see Table 1), the percentages of inhibition were determined at the inhibitor concentration equal to 9 µM and, for the most potent compounds, also at 900 nM, in the presence of 0.0833 U/mL of enzyme and 100 µM of acetylthiocholine as substrate.

In Table 1 the percentages of inhibition of amide derivatives **3**–**15** are reported. These data show that pyrimidine amide derivatives **3**–**11** are weak inhibitors of both enzymes, *Ee*AChE and *eq*BChE. If compared with the corresponding pyrimidine amino compounds previously investigated [32], it is evident that the replacement of the amino group with the amide has led to a drastic reduction in the inhibitory potency towards both cholinesterases. As an example, pyrimidine amide **3**, at a concentration equal to 9 µM, shows about 3% and 9% of inhibition, respectively, towards *Ee*AChE and *eq*BChE, while its corresponding diamine derivative, at the same concentration, showed 93% of inhibition towards *Ee*AChE and 26% towards *eq*BChE.

The pyridine amide derivatives **12**–**15** have percentages of inhibition significantly higher on both cholinesterases than the corresponding pyrimidine amide compounds **3–6**. In particular, these molecules are more potent towards *eq*BChE than towards *Ee*AChE. The comparison between pyridine amide **12** and the corresponding diamine compound [32] shows that, in this case, the replacement of the amino group with the amide reduced the inhibitory potency towards *Ee*AChE (73% for amine derivatives and 34% for **12**, both at 9 µM), but preserved the activity towards *eq*BChE (72% for amine derivatives and 81% for **12**, both at 9 µM), leading to a more selective BChE inhibitor. Among pyridine amides **12**–**15**, the most interesting for their inhibitory activity towards *eq*BChE are **12** and **14**, respectively, with a phenyl or 4-phenoxybenzyl ring adjacent to the carbonyl group. Compound **12** shows an 81% inhibition at 9 µM and 22% at 900 nM, while **14** has an 88% inhibition at 9 µM and 45% at 900 nM.

For carbamate derivatives **16**–**19**, whose data are reported in Table 2, a time-dependent inhibition assay was carried out to evaluate if they could act as carbamoylating inhibitor, forming a carbamoyl–cholinesterase complex by covalent interaction with the serine in the catalytic site. For this purpose, the percentages of inhibition were measured both at a time-point of zero and following an incubation of 1 h. An increase in the percentages of inhibition towards both cholinesterases was observed after the incubation period, suggesting that these compounds could act as carbamoylating agents. Moreover, these derivatives are more selective towards *eq*BChE than *Ee*AChE. In *eq*BChE, a more significant increase in the percentage of inhibition was observed following the incubation of the phenylcarbamates **16** and **18**, compared to the corresponding benzylcarbamates **17** and **19**; this could be explained by the fact that the phenate ion, being a better leaving group in comparison to the benzyl alcohol ion, can more easily allow the carbamoylation of the serine in the catalytic site. Moreover, in this case, as for amide derivatives, pyridine carbamates **18** and **19** show greater inhibitory potency on both cholinesterases, both at zero time and after incubation, compared to the corresponding pyrimidine derivatives **16** and **17**.

In general, these carbamates are better inhibitors of both cholinesterases if compared with the corresponding amide compounds. On the other hand, if compared with the corresponding pyrimidine and pyridine amino derivatives previously investigated [32], the replacement of the amino group with the carbamic one decreases the inhibitory potency towards *Ee*AChE but enhances it towards *eq*BChE, leading to more selective BChE inhibitors.

**Figure 2:**
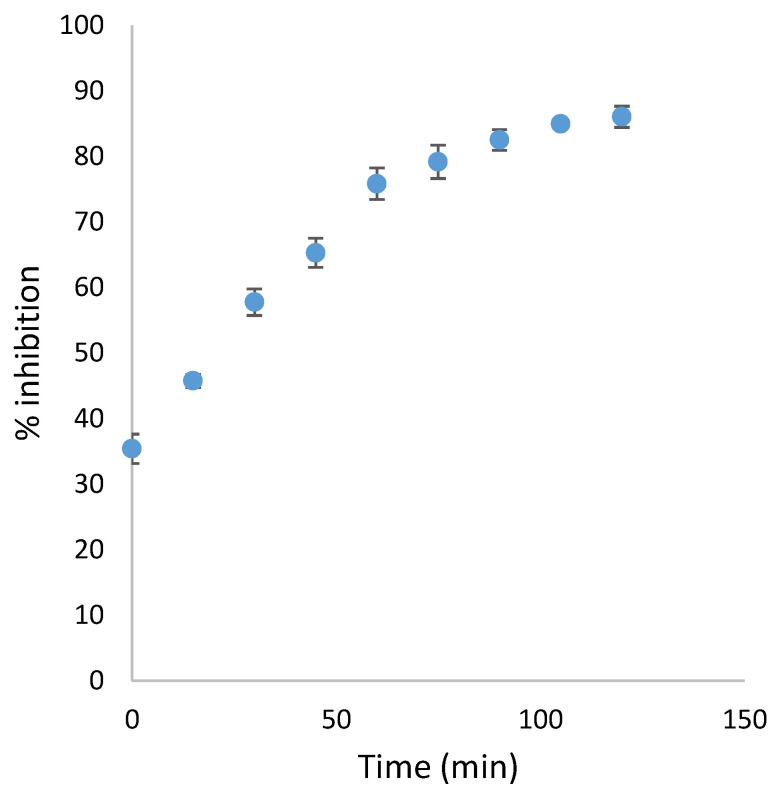
Plot of the percentage of inhibition exerted by carbamate **18** (700 nM) towards *eq*BChE (0.0833 U/mL), in presence of acetylthiocholine (100 µM), versus the incubation time.

To monitor the progressive increase over time of the *eq*BChE inhibition exerted by compound **18**, the most potent *eq*BChE inhibitor among these carbamate derivatives, the percentages of inhibition were determined every fifteen minutes for two hours, at the inhibitor concentration equal to 700 nM, in presence of 0.0833 U/mL of enzyme and 100 µM of acetylthiocholine. These inhibition percentage values were used to construct the graph related to the time-dependent inactivation of the *eq*BChE by carbamate **18**, reported below (Figure 2).

For compounds **12** and **14**, the most potent amide derivatives, such as the *eq*BChE inhibitors, the inhibition constant (K_i_) and the corresponding inhibition mechanism, were determined according to Dixon’s method [34], and the reciprocal of the hydrolysis rate versus the inhibitor concentrations at a fixed concentration of substrate were reported in graph. The recorded data were analysed with the enzyme kinetic module of SigmaPlot in order to find the best fitting model of inhibition, using the linear regression analysis. The reference kinetic models used in the regression analysis were: competitive, non-competitive, uncompetitive and mixed. Each determination was repeated five times and incorrect values were discarded to reduce the standard deviation to within the limit of 5%. In this way, the regression lines obtained have a linear regression coefficient (R^2^) higher than 0.98. The amide derivatives **12** and **14** revealed competitive inhibition mechanism towards *eq*BChE, with K_i_ respectively equal to 2.988 ± 0.190 µM and 0.621 ± 0.043 µM (Table 3). The Dixon’s plots of tested compounds are reported in the Supplementary Materials (Figures S1 and S2).

For pyridine phenylcarbamate **18** the IC_50_ on *eq*BChE, following incubation for one hour, was determined by virtue of a time dependent inhibition. The IC_50_ value was obtained by plotting the percentages of inhibition towards *eq*BChE versus the concentration of inhibitor expressed in logarithmic scale, at fixed substrate concentration (100 µM). The recorded data were analyzed with the enzyme kinetic module of SigmaPlot (Systat Software, Palo alto, CA, USA). Each measurement was replicated three times and the IC_50_ value obtained was confirmed by repeating the experiment twice. For compound **18** the IC_50_ on *eq*BChE is 454 ± 82 nM (Table 4). The IC_50_ plot of the tested compound is reported in the Supplementary Materials (Figure S3).

Moreover, it was evaluated whether the inhibition mechanism of carbamate **18** towards *eq*BChE is reversible, irreversible or pseudo-irreversible. For this purpose, the percentages of inhibition were determined both after one hour of the incubation of compound **18** (5.4 µM) with *eq*BChE (1.25 U), both following the removal of the inhibitor and the washing of the enzyme, carried out through ultrafiltration devices for the concentration of biological samples [35,36]. To validate the method, the same study was performed with carbaryl (54 µM), a known carbamoylating cholinesterase inhibitor.

After the incubation phase, the solution containing the inhibitor not bound to the enzyme was removed by centrifugation, retaining *eq*BChE on the membrane of the ultrafiltration devices. Then the enzyme was washed with phosphate buffer, to remove the bound inhibitor and to restore the enzyme activity. For carbamate **18**, a partial restoration of the enzymatic activity after washing was recorded, with a reduction in the percentage of inhibition from 89.7 to 56.7%. For carbaryl, a greater restoration of the enzymatic activity was recorded, with a reduction in the percentage of inhibition from 97.4 to 29.9%. Therefore, a pseudo-irreversible inhibition mechanism was observed for both compounds, with the difference that the decarbamoylation phase for carbaryl is more rapid than for carbamate **18**.

### 2.3. In Silico Studies

Computational approach was performed to gain structural insights into the binding mode of pyrimidine and pyridine amide and carbamate derivatives to human AChE (*h*AChE) and human BChE (*h*BChE). For the current analysis, the recent X-ray crystallographic structures of the *h*AChE (PDB code: 6ZWE) and the *h*BChE (PDB code: 6ZWI) in complex with a triphenylphosphonium conjugate were used [37].

Even if the apo-crystal structures of *Ee*AChE are deposited in the protein data bank (PDB), they cannot be considered to be good quality models. By superposing the *Ee*AChE (PDB code: 1C2O) and *h*AChE structures (PDB code: 4EY7) with the model (PDB code: 6ZWE), the root-mean-square deviation (RMSD) values of 0.79 and 0.55 Å, respectively, were obtained (Supplementary Materials, Figure S4a), thus providing a reliable *h*AChE receptor for the molecular recognition analysis. Instead, regarding the choice of the BChE receptor, no crystal structure of *eq*BChE was available in PDB. The sequence of *eq*BChE derived from the Uniprot Database shares 90% sequence identity with that of the adopted *h*BChE, above all comparing residues of the binding site (Figure S5). By the overlapping of the X-ray structure of the previous work (PDB code: 5NN0) and the selected model (PDB code: 6ZWI) (Figure S4b), it was found the RMSD value equal to 0.26 Å, so justifying the selection of *h*BChE for the computational study. According to the criteria for the identification of reliable complexes reported in the literature [38], and the availability of the resolved crystal structures of both enzymes in the same experimental conditions, these models were considered as a good starting point for a better comparison by in silico approaches.

**Figure 3:**
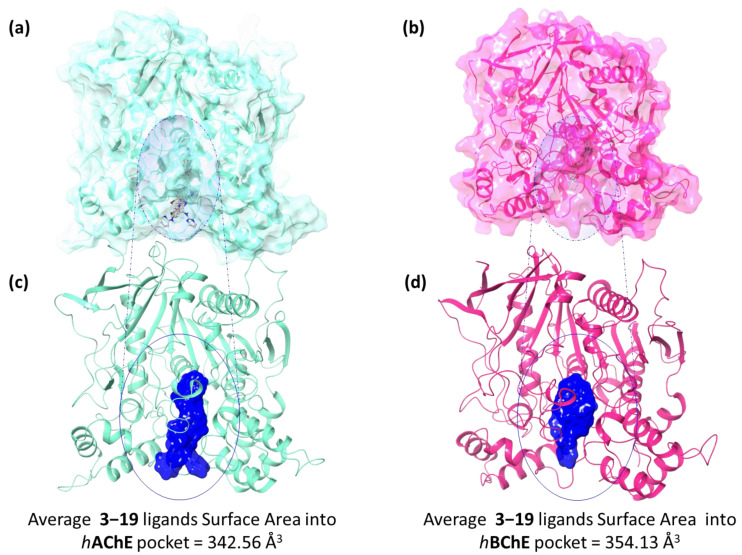
Superposition of all synthesized ligands into (**a**) *h*AChE and (**b**) *h*BChE binding pocket, surrounded by a blue circle. The ligands are illustrated as grey carbon ball and sticks representations. Panels (**c**,**d**) are focused on the solid ligand surface (**blue**) occupied by all compounds in *h*AChE and *h*BChE, respectively. The average **3**–**19** ligands’ surface area in both the *h*AChE and *h*BChE pocket are reported below each 3D representation.

The molecular recognition protocol was validated by docking cocrystallized ligands into the binding site. The RMSD values between the native pose of cocrystallized ligands against *h*AChE and *h*BChE and the related best redocked conformations were found to be 0.96 Å and 0.76 Å, respectively, thus revealing the assurance of docking protocol (Figure S6). According with previously published studies [31,32,39] no linear correlation between docking score and the experimental data is expected (Table S1). Despite the structural differences of the two ChEs cavities, the alkyl linker of six methylene units between the pyrimidine or pyridine moiety and the amide or carbamate group of the **3–19** compounds ensured the appropriate flexibility to accommodate into the three domains lining the gorge of the ChEs. Indeed, analysing the best docking poses, all compounds well recognized both the pockets of the enzymes. The average molecular surface area of all docked ligands, calculated by using the “ink-blot” method, is equal to 342.56 Å^3^ and 354.13 Å^3^, into *h*AChE (Figure 3a,c) and *h*BChE (Figure 3b,d) receptors, respectively (Table S2).

Taking into account the obtained experimental data, we performed the molecular recognition of compounds **12**, **14** and **18** towards the two ChEs receptors in order to better understand their binding modes. 

**Figure 4:**
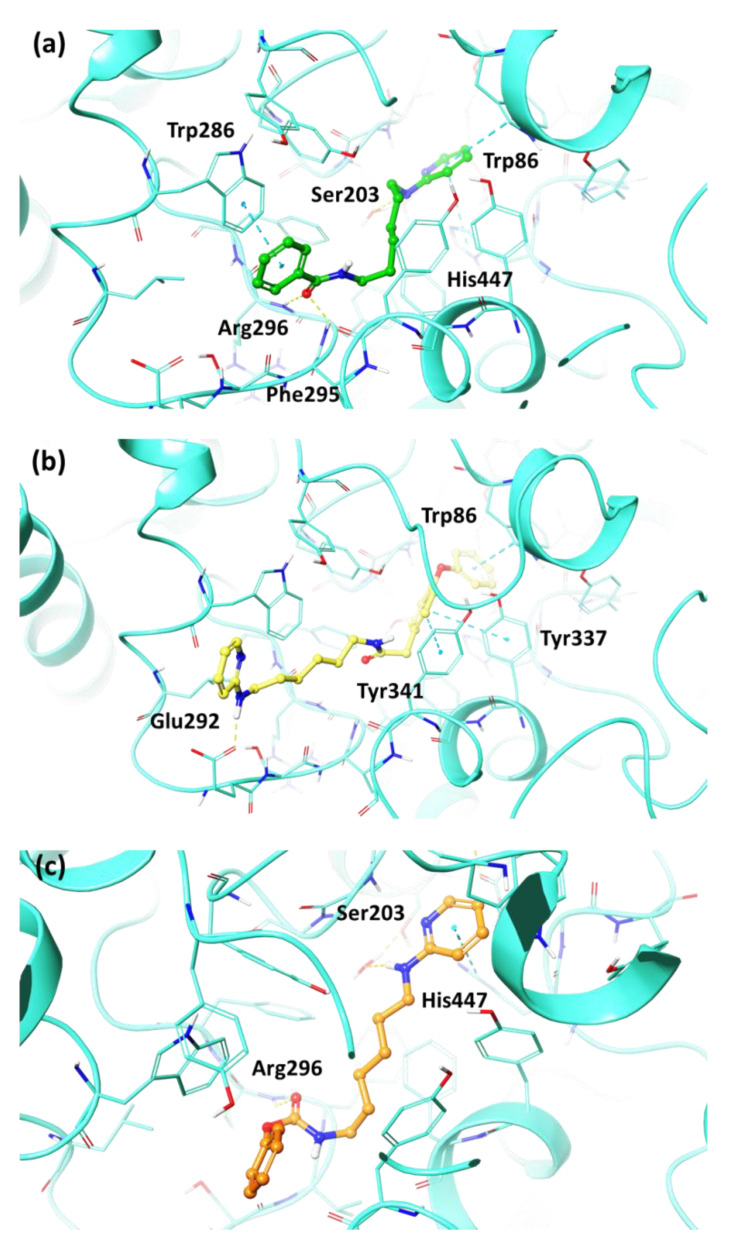
Best docking pose of compounds (**a**) **12**, (**b**) **14**, and (**c**) **18** into the *h*AChE active site shown as wire representation. Most relevant interacting residues are displayed as thin turquoise tubes. Compounds **12**, **14** and **18** are illustrated in green, yellow and orange carbon ball and stick representations, respectively. Hydrogen bonds and π–π interactions are, respectively, represented in yellow and blue dotted lines.

Regarding *h*AChE, the pyridine group of the compound **12** (Figure 4a) formed two π–π stacking interactions with His447 and Trp86, while the amine reached Ser203 through a water bridge. Instead, the phenyl ring bound Trp286 by a π–π stacking and the amide portion contributed to the accommodation at the mid-gorge pocket by means of two H-bonds with Phe295 and Arg296 backbone atoms. The growth size of the compound **14** due to the presence of the 4-phenoxybenzyl ring (Figure 4b) led to an opposite orientation with respect to the others binding poses, characterized by three π–π stacking interactions with Trp86, Tyr337 and Tyr341. Conversely, the amine group engaged a H-bond with Glu292. Concerning compound **18** (Figure 4c), the carbamate group pointed towards the backbone of Arg296, the pyridine moiety was stabilized by a π–π stacking interaction with His447 and the amine portion interacted with Ser203 by means of a water bridge.

Analysing the behaviour of these three compounds towards *h*BChE enzyme, an increased theoretical binding affinity for compounds **12**, **14** and **18** to *h*BChE with respect to the *h*AChE pocket was observed. These findings were associated with the presence of a more hydrophilic and charged region in the *h*BChE binding site, which probably led to the selectivity in regard to *h*BChE (Figure S7).

**Figure 5:**
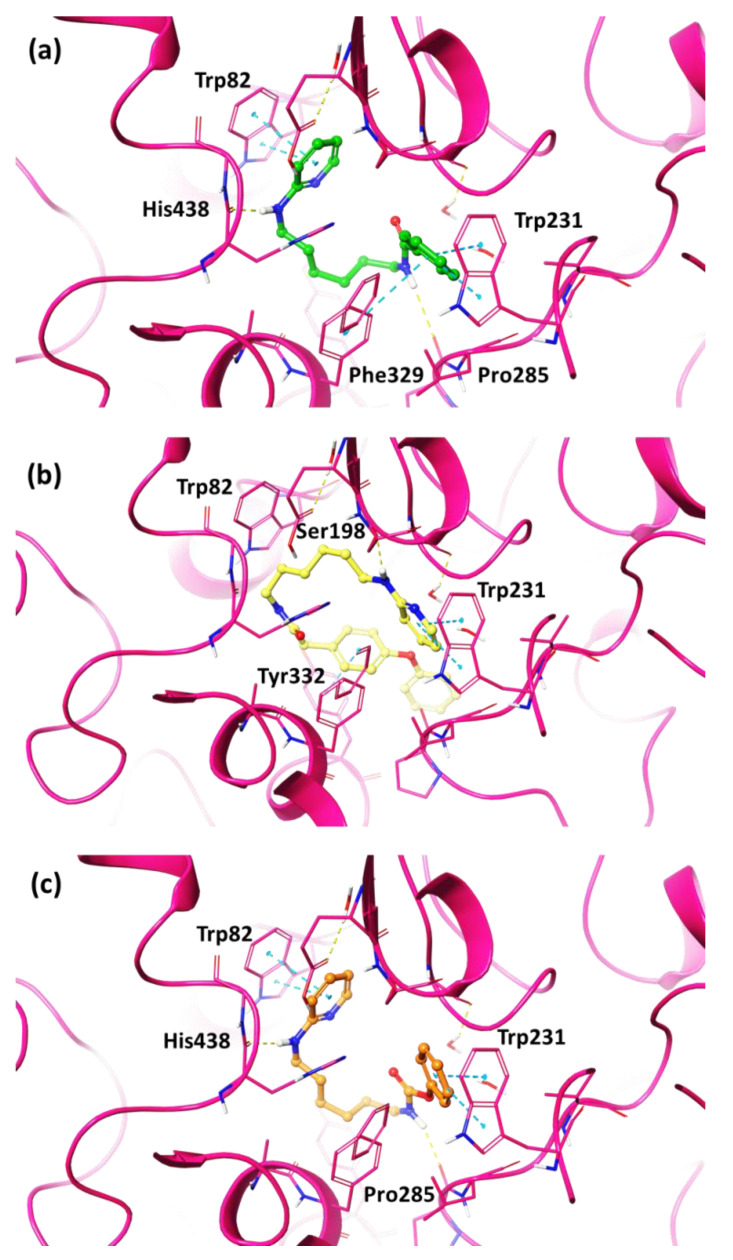
Best docking pose of compounds (**a**) **12**, (**b**) **14**, and (**c**) **18** into the *h*BChE active site shown as wire representation. Most relevant interacting residues are displayed as thin pink tubes. Compounds **12**, **14** and **18** are illustrated in green, yellow and orange carbon ball and stick representations, respectively. Hydrogen bonds and π–π interactions are, respectively, represented in yellow and blue dotted lines.

Specifically, the pyridine moiety of compound **12** (Figure 5a) created a π–π interaction with Trp82, while the amine portion engaged a hydrogen bond with His438. Furthermore, two π–π interactions were formed between the phenyl group and Trp231 and Phe329 residues, and an additional H-bond between the *N* atom of the amide and Pro285 backbone was observed.

Compound **14** (Figure 5b) interacted with Tyr332 by means of the 4-phenoxybenzyl ring, while the pyridine group pointed to Trp231 in CAS region. Its ammine portion was able to engage a H-bond with Ser198.

As regards the compound **18**, the most interesting among the carbamate derivatives, the pyridine moiety established a π–π interaction with Trp82, the amino group engaged a H-bond with His438, while the phenylcarbamate portion formed a closer network of interactions with Trp231 and Pro285 (Figure 5c).

### 2.4. Molecular Dynamics Studies

**Figure 6:**
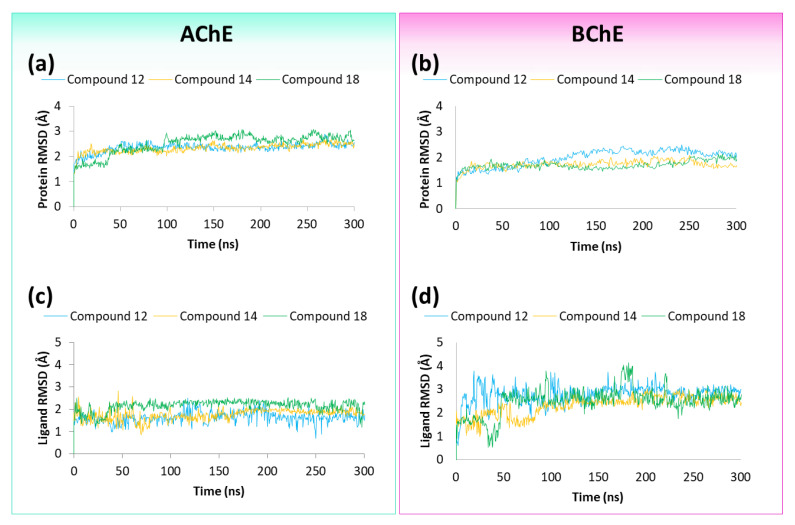
Protein RMSD and Ligand RMSD plots of compounds **12**, **14** and **18** to (**a**,**c**) *h*AChE and (**b**,**d**) *h*BChE, respectively.

The best docked poses of compounds **12**, **14** and **18** into the binding pockets of both *h*AChE and *h*BChE were submitted to 300 ns of molecular dynamics (MDs) simulations. The RMSD of both the proteins’ Cα atoms and the ligands were calculated in order to evaluate the structural conformational changes of the protein with respect to the initial frame and the stability of the ligand, in regard to both the receptors and their binding pockets (Figure 6). As shown, the RMSD plot indicated that the three compounds in complex to *h*AChE (Figure 6a) and *h*BChE (Figure 6b) remained stable during the whole MDs, thus ensuring a good equilibrium for the systems. Furthermore, *h*AChE, in complex with compounds **12**, **14** and **18,** was associated with a decreased conformational stability with RMSD average values of 2.35, 2.31 and 2.50 Å, respectively, if compared to the behaviour of *h*BChE complexed with compound **12** (2.15 Å), **14** (1.71 Å) and **18** (1.88 Å). The analysis of the ligands’ RMSD trend revealed that compound **12** and **18** were able to better maintain their binding mode during the whole MDs, respectively, into *h*AChE (Figure 6c) and *h*BChE (Figure 6d), in comparison to the other analysed compounds. 

In order to obtain further insights about the selectivity of the compounds **12**, **14** and **18** towards BChE with respect to AChE, the single contributions of hydrophobic, water bridge and hydrogen-bonding interactions, pivotal interactions into ChEs active sites, were considered.

In detail, regarding the *h*AChE enzyme, it was observed that the compound **12** benzamido group interacted mainly with Phe295 for 32% of the MDs time by means a water bridge, while engaged hydrophobic contacts with Trp86, Trp286 and Tyr441 residues. The presence of a 4-phenoxybenzyl ring led to compound **14** being well accommodated into CAS and mid-gorge pockets thus ensuring several interactions in the enzyme. Indeed, during the MDs, the amide group provided a H-bond with Tyr124 (79%) at the CAS site and a water bridge with Phe295 (60%), located at the mid-gorge region. The amino portion was directly related to Ser293 (30%), while the 4-phenoxybenzyl ring engaged a π–π stacking interaction with Tyr337 for half of the MDs. Finally, the amino group and the phenylcarbamate of compound **18** participated in major interactions with His447 (36%) and Phe295 (75%), respectively.

Analysing the dynamic behaviour of the investigated compounds towards the *h*BChE enzyme, during 300 ns of MDs, it was observed that in compound **12,** the benzamido group was anchored to Ser287 (43%), Trp231 (37%) and Phe329 (81%) in the CAS and mid-gorge pockets. However, the amino-pyridine portion engaged in a hydrophobic interaction with Phe73 (25%) and an H-bond with Thr284 (37%), respectively, in PAS and mid-gorge sites. The steric hindrance brought about by the 4-phenoxybenzyl ring in compound **14** allowed it to orient the pyridine moiety versus the catalytic region, which established a water bridge with His438 (53%) and a π–π stacking interaction with Trp231 (79%), whilst the amino group was able to form a hydrogen bond with Pro285 (67%) in the CAS pocket. Lastly, the phenylcarbamate moiety of compound **18** engaged hydrophobic interactions with Trp231 (53%) and a H-bond with Ser287 (75%). Presumably, this pivotal interaction in the catalytic site could represent the main reason for the selectivity of compound **18** towards BChE, carrying it effortlessly to the carbamoylation of the serine.

Computational analysis revealed that the multiple interactions of compound **12**, **14** and **18** with specific gorge hot spots may trap the enzyme in a peculiar conformation, decreasing the ability of the enzyme-inhibitor complex to access protein fluctuations, thus improving the compounds inhibitory potency. Taking into account that the conformational heterogeneity in ChEs is related to specific classical or non-classical functions, a promising inhibitor should be able to freeze the “classical” enzyme structure in a particular conformational state, thus preventing acetylcholine hydrolysis and non-classical protein functions. Our results may confirm that the pyridine moiety placed into the alkyl chain of six methylene units represents a crucial pharmacophore feature for ChEs inhibition, as detected in our previous work [32]. Still, the stronger inhibition capability of compound **18** may suggest that the presence of phenylcarbamate insertion is more suitable for BChE enzyme interaction, ensuring the selectivity toward its binding pocket.

### 2.5. Chelation Studies

For compounds **12**, **14** and **18**, the most potent as inhibitors of *eq*BChE, the chelating ability on the biometals Fe^3+^, Cu^2+^ and Zn^2+^ was evaluated, in order to define their possible multifunctional profile.

Initially, the UV–Vis spectrum of the ligand was recorded and compared with the spectra obtained by adding an excess of metal to the ligand solution, maintaining the same concentration of the ligand (ligand/metal ratio 1:3, 1:5 or 1:10). The variation of the UV–Vis spectrum of the ligand in presence of metal ions is indicative of the formation of the complex. Based on this, it has been observed that all the tested pyridine derivatives have the ability to chelate Fe^3+^ and Cu^2+^ ions but not Zn^2+^. As previously supposed, the chelating activity of these compounds is due to the presence of two adjacent nitrogen atoms in the 2-amino-pyridine moiety.

**Figure 7:**
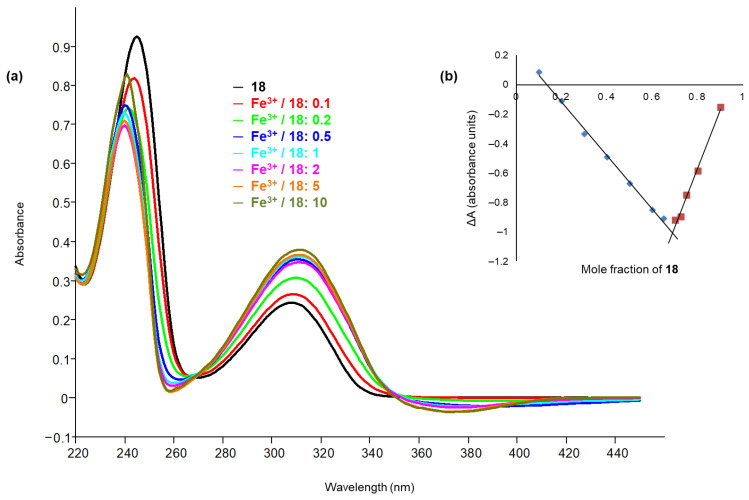
(**a**) UV–Vis titration of ligand **18** with Fe^3+^. The greatest variations in spectra with increasing amount of metal are observed at 245 nm, with a reduction of absorbance, and at 310 nm, with an increase of absorbance. There are two isosbestic points at 267 and 353 nm. (**b**) Job’s plot of compound **18** in presence of Fe^3+^: variation of the absorbance (ΔA) at the wavelength of 245 nm, in ordinate, versus the mole fraction of **18**, in abscissa. X (mole fraction that causes the maximum variation of absorbance) = 0.69; n (number of ligand molecules per cation) = 2.

UV–Vis titrations of these compounds were carried out with the metal ions with which they form complexes, recording first the UV–Vis spectrum of the ligand and then the spectra obtained by mixing solutions of ligand and metal according to increasing metal/ligand molar ratios (Figure 7a).

The stoichiometries of metal–ligand complexes were determined through Job’s method [40,41].

In the Job’s plot, the values of ΔA, measured at the selected wavelengths, where evident absorbance variations were observed in the titration spectra, are in ordinate and the mole fractions of the ligand are in abscissa. The mole fraction X, which causes the maximum variation of absorbance, is extracted from the graph and then the value of the coefficient n, which corresponds to the number of ligand molecules per cation, is obtained (Figure 7b). The UV–Vis titrations spectra and the Job’s plots of all tested compound are reported in the Supplementary Materials (Figures S8–S17), while the data extrapolated from Job’s plots are summarized in Table 5. 

### 2.6. Inhibition of Amyloid and Tau Aggregation

For compounds **12**, **14** and **18,** the inhibitory activity against Aβ_42_ and tau aggregation was evaluated. The anti-aggregating effect of tested compounds was monitored by a cell-based assay in intact *Escherichia coli* cells that overexpress either Aβ_42_ peptide or tau protein, which, upon expression, form insoluble inclusion bodies that were stained with thioflavin-S [42]. The percentages of inhibition towards Aβ_42_ and tau aggregation were determined at an inhibitor concentration of 100 µM and the obtained results are reported in Table 6. In general, these compounds show a weak anti-aggregating activity; among these, the pyridine carbamate **18** is the best inhibitor of aggregation of both Aβ_42_ and tau.

### 2.7. Computation of Physicochemical Descriptors and ADME Parameters

Physicochemical descriptors and ADME parameters of compounds **12**, **14** and **18**, the most interesting by virtue of their inhibitory activity towards BChE, as well as their chelating ability, were predicted by means of the SwissADME public server [43] and the obtained data are reported in Table 7. All studied compounds fit the Lipinski’s rule of five (MW ≤ 500; MLogP ≤ 4.15; H bond acceptor ≤ 10; H bond donor ≤ 5) [44].

They should be soluble or moderately soluble in water. Moreover, they should have high gastrointestinal absorption after oral administration, and they should be BBB accessible.

### 2.8. Cell Viability Studies

**Figure 8:**
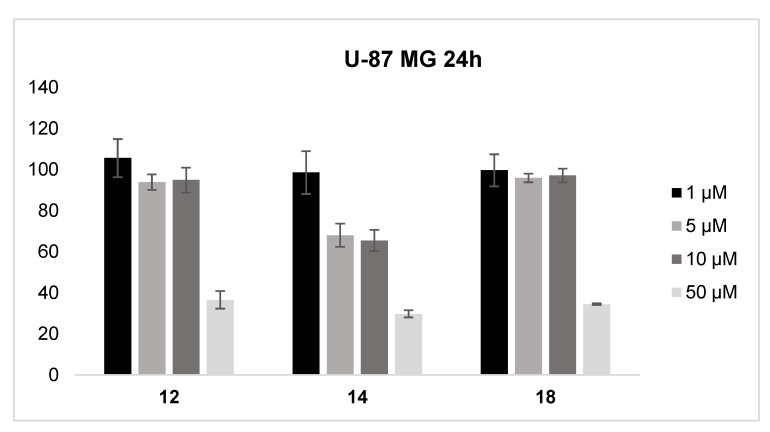
Cell viability assay of U-87 MG cells in the presence of an increasing concentration of the studied compounds was evaluated using the MTT method. Data represent the absorbance relative to untreated cells in the same experiment and standardized to 100%. All data points were performed in triplicate and at least in three independent experiments.

Compounds **12**, **14** and **18** were tested to evaluate the cytotoxic effects on U-87 MG Cell Line from human brain (glioblastoma astrocytoma) at concentration ranging from 1 to 50 µM. The obtained data, represented in the histogram of Figure 8, suggest that the tested compounds are characterized by low toxicity towards the studied cells; in particular, compounds **12** and **18** showed a similar dose related toxicity profile, providing a cell viability over 90% up to a concentration equal to 10 µM and above 35% at 50 µM; instead, compound **14** showed a cell viability above 65% at 10 µM and 30% at 50 µM.

## 3. Materials and Methods

### 3.1. Chemistry

All reagents and solvents were of analytical grade and were purchased from Sigma-Aldrich (Milano, Italy) or from Fluorochem (Hadfield, UK). Triethylamine was freshly purified by distillation over potassium hydroxide. Column chromatographies were performed on silica gel (Merck; 63−200 μm particle size). ^1^H-NMR and ^13^C-NMR spectra were acquired at 25 °C on a Bruker AVANCE-400 spectrometer at 9.4 Tesla operating at 400 MHz (^1^H-NMR) and 100 MHz (^13^C-NMR) or on a Bruker AVANCE-200 spectrometer at 4.7 Tesla operating at 50 MHz (^13^C-NMR) (Bruker, Billerica, MA, USA); chemical shift values (δ) are given in ppm, relative to TMS, using the solvent as the internal reference; coupling constants are given in Hz. The following abbreviation were used: s = singlet, d = doublet, t = triplet, q = quartet, dd = double doublet, ddd = double double doublet, dt = double triplet, bs = broad singlet, bt = broad triplet, m = multiplet. Mass spectra were recorded on a ThermoFinnigan LCQ Classic (San Jose, CA, USA) LC/MS/MS ion trap equipped with an ESI source and a syringe pump; samples (10^−4^–10^−5^ M in MeOH/H_2_O 80:20) were infused in the electrospray system at a flow rate of 5–10 µL·min^−1^; when necessary, 50 µL of 10^−2^ M HCOOH or 10^−2^ M NH_3_ were added to the sample solutions, in order to promote the analyte ionisation; the ESI-MS data are given as *m*/*z*, with mass expressed in amu. Melting points were determined on a FALC Mod. 360 D apparatus and are uncorrected. Infrared spectra were recorded on a Perkin Elmer Spectrum One FT-IR spectrometer equipped with an ATR system. The purity of the compounds was determined by elemental analyses obtained by a PE 2400 (Perkin-Elmer, Waltham, MA, USA) analyzer and the analytical results were within ±0.4% of the theoretical values for all compounds.

#### 3.1.1. General Procedure for the Synthesis of Pyrimidine and Pyridine Amide Derivatives **3**–**15**

To a solution of 1,1-carbonyldiimidazole (1 eq) in 15 mL of CH_3_CN the opportune carboxylic acid (1 eq) was added and the reaction mixture was stirred under reflux conditions for 5 h. Then intermediate **1** or **2** (1 eq), obtained as previously reported [31], was dissolved in 10 mL of warm CH_3_CN and added to the reaction mixture, which was then refluxed for 18 h. After this time, the solvent was removed under reduced pressure. The obtained residue was purified by column chromatography on silica gel and/or by crystallisation.

*N*-(6-(pyrimidin-2-ylamino)hexyl)benzamide (**3**)

Compound **3** was prepared using 1,1-carbonyldiimidazole (0.081 g, 0.50 mmol), benzoic acid (0.061 g, 0.50 mmol), *N*^1^-(pyrimidin-2-yl)hexane-1,6-diamine (**1**) (0.097 g, 0.50 mmol), following the general procedure described above. Column chromatography: silica gel, CH_2_Cl_2_/isopropanol 9:1, R_f_ = 0.55. White solid, 0.119 g, 80% yield.

^1^H-NMR (400 MHz) (CD_3_CN) δ (ppm): 8.21 (d, 2H, *J* = 4.72 Hz, pyrimidine); 7.79–7.76 (m, 2H, aromatic); 7.53–7.49 (m, 1H, aromatic); 7.46–7.42 (m, 2H, aromatic); 7.02 (bs, 1H, -NH-C=O); 6.50 (t, 1H, *J* = 4.76 Hz, pyrimidine); 5.71 (bs, 1H, pyrimidine-NH-); 3.36–3.30 (m, 4H, -NH-CH_2_-CH_2_-CH_2_-CH_2_-CH_2_-CH_2_-NH-); 1.61–1.54 (m, 4H, -NH-CH_2_-CH_2_-CH_2_-CH_2_-CH_2_-CH_2_-NH-); 1.41–1.38 (m, 4H, -NH-CH_2_-CH_2_-CH_2_-CH_2_-CH_2_-CH_2_-NH-). ^13^C-NMR (100 MHz) (MeOD) δ (ppm): 170.2; 163.5; 159.2; 135.9; 132.5; 129.5; 128.2; 110.9; 42.1; 41.0; 30.5; 30.4; 27.9; 27.7. ESI-MS (*m/z*): [M+H]^+^ = 299.02. Anal. (C_17_H_22_N_4_O) C, H, N Calcd: C 68.43%, H 7.43%, N 18.78%; Found: C 68.36%, H 7.44%, N 18.83%. m.p.: 112–114 °C.

2-phenyl-*N*-(6-(pyrimidin-2-ylamino)hexyl)acetamide (**4**)

Compound **4** was prepared using 1,1-carbonyldiimidazole (0.081 g, 0.50 mmol), phenylacetic acid (0.068 g, 0.50 mmol), *N*^1^-(pyrimidin-2-yl)hexane-1,6-diamine (**1**) (0.097 g, 0.50 mmol), following the general procedure described above. Column chromatography: silica gel, CH_2_Cl_2_/MeOH 8.5:1.5, R_f_ = 0.72. White solid, 0.138 g, 88% yield.

^1^H-NMR (400 MHz) (CD_3_CN) δ (ppm): 8.22 (d, 2H, *J* = 4.72 Hz, pyrimidine); 7.33–7.22 (m, 5H, aromatic); 6.51 (t, 1H, *J* = 4.76 Hz, pyrimidine); 6.42 (bs, 1H, -NH-C=O); 5.68 (bs, 1H, pyrimidine-NH-); 3.41 (s, 2H, Ar-CH_2_-C=O); 3.30 (q, 2H, *J* = 6.92 Hz, pyrimidine-NH-CH_2_-); 3.10 (q, 2H, *J* = 6.76 Hz, -CH_2_-NH-C=O); 1.57–1.49 (m, 2H, -NH-CH_2_-CH_2_-); 1.46–1.39 (m, 2H, -NH-CH_2_-CH_2_-); 1.37–1.24 (m, 4H, -NH-CH_2_-CH_2_-CH_2_-CH_2_-CH_2_-CH_2_-NH-). ^13^C-NMR (100 MHz) (MeOD) δ (ppm): 173.9; 163.5; 159.2; 137.1; 130.0; 129.5; 127.8; 110.9; 43.9; 42.1; 40.4; 30.32; 30.27; 27.63; 27.61. ESI-MS (*m*/*z*): [M+H]^+^ = 313.06. Anal. (C_18_H_24_N_4_O) C, H, N Calcd: C 69.20%, H 7.74%, N 17.93%; Found: C 69.23%, H 7.76%, N 17.87%. m.p.: 91–93 °C.

2-(4-phenoxyphenyl)-*N*-(6-(pyrimidin-2-ylamino)hexyl)acetamide (**5**)

Compound **5** was prepared using 1,1-carbonyldiimidazole (0.058 g, 0.36 mmol), (4-phenoxyphenyl)acetic acid (0.082 g, 0.36 mmol), *N*^1^-(pyrimidin-2-yl)hexane-1,6-diamine (**1**) (0.070 g, 0.36 mmol), following the general procedure described above. Column chromatography: silica gel, CH_2_Cl_2_/isopropanol 9.5:0.5, R_f_ = 0.45. White solid, 0.077 g, 53% yield.

^1^H-NMR (400 MHz) (CD_3_CN) δ (ppm): 8.21 (d, 2H, *J* = 4.76 Hz, pyrimidine); 7.39–7.34 (m, 2H, aromatic); 7.27–7.24 (m, 2H, aromatic); 7.14–7.10 (m, 1H, aromatic); 7.00–6.97 (m, 2H, aromatic); 6.96–6.92 (m, 2H, aromatic); 6.50 (t, 1H, *J* = 4.76 Hz, pyrimidine); 6.45 (bs, 1H, -NH-C=O); 5.69 (bs, 1H, pyrimidine-NH-); 3.40 (s, 2H, Ar-CH_2_-C=O); 3.30 (q, 2H, *J* = 6.92 Hz, pyrimidine-NH-CH_2_-); 3.11 (q, 2H, *J* = 6.80 Hz, -CH_2_-NH-C=O); 1.57–1.50 (m, 2H, -NH-CH_2_-CH_2_-); 1.47–1.40 (m, 2H, -NH-CH_2_-CH_2_-); 1.38–1.25 (m, 4H, -NH-CH_2_-CH_2_-CH_2_-CH_2_-CH_2_-CH_2_-NH-). ^13^C-NMR (100 MHz) (MeOD) δ (ppm): 174.0; 163.5; 159.2; 158.8; 157.6; 132.1; 131.5; 130.8; 124.3; 119.9; 119.7; 111.0; 43.1; 42.1; 40.5; 30.4; 30.3; 27.64; 27.62. ESI-MS (*m*/*z*): [M+H]^+^ = 404.80. Anal. (C_24_H_28_N_4_O_2_) C, H, N Calcd: C 71.26%, H 6.98%, N 13.85%; Found: C 71.23%, H 6.99%, N 13.89%. m.p.: 98–99 °C.

*N*-(6-(pyrimidin-2-ylamino)hexyl)cinnamamide (**6**)

Compound **6** was prepared using 1,1-carbonyldiimidazole (0.088 g, 0.54 mmol), *trans*-cinnamic acid (0.080 g, 0.54 mmol), *N*^1^-(pyrimidin-2-yl)hexane-1,6-diamine (**1**) (0.105 g, 0.54 mmol), following the general procedure described above. Column chromatography: silica gel, CH_2_Cl_2_/hexane/isopropanol 9:1:0.5, R_f_ = 0.38. White solid, 0.112 g, 64% yield.

^1^H-NMR (400 MHz) (CD_3_CN) δ (ppm): 8.22 (d, 2H, *J* = 4.76 Hz, pyrimidine); 7.57–7.55 (m, 2H, aromatic); 7.48 (d, 1H, *J* = 15.72 Hz, Ar-CH=CH-); 7.42–7.34 (m, 3H, aromatic); 6.61 (bs, 1H, -NH-C=O); 6.56–6.49 (m, 2H, Ar-CH=CH- and pyrimidine); 5.70 (bs, 1H, pyrimidine-NH-); 3.33 (q, 2H, *J* = 6.84 Hz, pyrimidine-NH-CH_2_-); 3.25 (q, 2H, *J* = 6.84 Hz, -CH_2_-NH-C=O); 1.61–1.48 (m, 4H, -NH-CH_2_-CH_2_-CH_2_-CH_2_-CH_2_-CH_2_-NH-); 1.39–1.37 (m, 4H, -NH-CH_2_-CH_2_-CH_2_-CH_2_-CH_2_-CH_2_-NH-). ^13^C-NMR (100 MHz) (*d_6_*-DMSO) δ (ppm): 164.9; 161.4; 157.8; 138.4; 135.0; 129.4; 129.0; 127.5; 122.4; 109.6; 40.6; 38.7; 29.2; 28.8; 26.3; 26.2. ESI-MS (*m*/*z*): [M+H]^+^ = 324.7. Anal. (C_19_H_24_N_4_O) C, H, N Calcd: C 70.34%, H 7.46%, N 17.27%; Found: C 70.42%, H 7.42%, N 17.23%. m.p.: 94–95 °C.

4-bromo-*N*-(6-(pyrimidin-2-ylamino)hexyl)benzamide (**7**)

Compound **7** was prepared using 1,1-carbonyldiimidazole (0.065 g, 0.40 mmol), 4-bromobenzoic acid (0.080 g, 0.40 mmol), *N*^1^-(pyrimidin-2-yl)hexane-1,6-diamine (**1**) (0.078 g, 0.40 mmol), following the general procedure described above. Column chromatography: silica gel, CH_2_Cl_2_/isopropanol 9:1, R_f_ = 0.5. White solid, 0.113 g, 72% yield.

^1^H-NMR (400 MHz) (CD_3_CN) δ (ppm): 8.21 (d, 2H, *J* = 4.76 Hz, pyrimidine); 7.70–7.67 (m, 2H, aromatic); 7.62–7.59 (m, 2H, aromatic); 7.05 (bs, 1H, -NH-C=O); 6.50 (t, 1H, *J* = 4.76 Hz, pyrimidine); 5.69 (bs, 1H, pyrimidine-NH-); 3.32 (q, 4H, *J* = 6.80 Hz, -NH-CH_2_-CH_2_-CH_2_-CH_2_-CH_2_-CH_2_-NH-); 1.61–1.54 (m, 4H, -NH-CH_2_-CH_2_-CH_2_-CH_2_-CH_2_-CH_2_-NH-); 1.41–1.37 (m, 4H, -NH-CH_2_-CH_2_-CH_2_-CH_2_-CH_2_-CH_2_-NH-). ^13^C-NMR (100 MHz) (CDCl_3_) δ (ppm): 166.7; 161.8; 157.9; 133.7; 131.9; 128.7; 126.1; 110.3; 41.4; 40.1; 29.6; 29.5; 26.7; 26.6. ESI-MS (*m*/*z*): [M+H]^+^ = 376.93 (98), 378.93 (100). Anal. (C_17_H_21_BrN_4_O) C, H, N Calcd: C 54.12%, H 5.61%, N 14.85%; Found: C 54.20%, H 5.59%, N 14.81%. m.p.: 139–141 °C.

4-chloro-*N*-(6-(pyrimidin-2-ylamino)hexyl)benzamide (**8**)

Compound **8** was prepared using 1,1-carbonyldiimidazole (0.065 g, 0.40 mmol), 4-chlorobenzoic acid (0.063 g, 0.40 mmol), *N*^1^-(pyrimidin-2-yl)hexane-1,6-diamine (**1**) (0.078 g, 0.40 mmol), following the general procedure described above. Column chromatography: silica gel, CH_2_Cl_2_/MeOH 9.5:0.5, R_f_ = 0.57. White solid, 0.092 g, 69% yield.

^1^H-NMR (400 MHz) (CD_3_CN) δ (ppm): 8.21 (d, 2H, *J* = 4.72 Hz, pyrimidine); 7.77–7.74 (m, 2H, aromatic); 7.47–7.43 (m, 2H, aromatic); 7.06 (bs, 1H, -NH-C=O); 6.50 (t, 1H, *J* = 4.76 Hz, pyrimidine); 5.70 (bs, 1H, pyrimidine-NH-); 3.32 (q, 4H, *J* = 6.64 Hz, -NH-CH_2_-CH_2_-CH_2_-CH_2_-CH_2_-CH_2_-NH-); 1.61–1.54 (m, 4H, -NH-CH_2_-CH_2_-CH_2_-CH_2_-CH_2_-CH_2_-NH-); 1.44–1.35 (m, 4H, -NH-CH_2_-CH_2_-CH_2_-CH_2_-CH_2_-CH_2_-NH-). ^13^C-NMR (100 MHz) (MeOD) δ (ppm): 169.0; 163.5; 159.2; 138.6; 134.5; 129.9; 129.7; 111.0; 42.1; 41.0; 30.41; 30.37; 27.8; 27.7. ESI-MS (*m*/*z*): [M+H]^+^ = 332.97 (100), 334.90 (36). Anal. (C_17_H_21_ClN_4_O) C, H, N Calcd: C 61.35%, H 6.36%, N 16.83%; Found: C 61.26%, H 6.37%, N 16.87%. m.p.: 124–126 °C.

2-methoxy-*N*-(6-(pyrimidin-2-ylamino)hexyl)benzamide (**9**)

Compound **9** was prepared using 1,1-carbonyldiimidazole (0.058 g, 0.36 mmol), 2-methoxybenzoic acid (0.055 g, 0.36 mmol), *N*^1^-(pyrimidin-2-yl)hexane-1,6-diamine (**1**) (0.070 g, 0.36 mmol), following the general procedure described above. Column chromatography: silica gel, CH_2_Cl_2_/isopropanol 9.5:0.5, R_f_ = 0.32. White solid, 0.077 g, 65% yield.

^1^H-NMR (400 MHz) (CD_3_CN) δ (ppm): 8.21 (d, 2H, *J* = 4.76 Hz, pyrimidine); 7.97 (dd, 1H, *J_1_* = 7.72 Hz, *J_2_* = 1.80 Hz, aromatic); 7.88 (bs, 1H, -NH-C=O); 7.49–7.44 (m, 1H, aromatic); 7.10–7.02 (m, 2H, aromatic); 6.50 (t, 1H, *J* = 4.76 Hz, pyrimidine); 5.74 (bs, 1H, pyrimidine-NH-); 3.93 (s, 3H, Ar-OCH_3_); 3.38–3.30 (m, 4H, -NH-CH_2_-CH_2_-CH_2_-CH_2_-CH_2_-CH_2_-NH-); 1.61–1.54 (m, 4H, -NH-CH_2_-CH_2_-CH_2_-CH_2_-CH_2_-CH_2_-NH-); 1.41–1.38 (m, 4H, -NH-CH_2_-CH_2_-CH_2_-CH_2_-CH_2_-CH_2_-NH-). ^13^C-NMR (100 MHz) (MeOD) δ (ppm): 168.3; 163.6; 159.2; 159.0; 133.9; 131.9; 123.5; 121.9; 112.8; 111.0; 56.5; 42.1; 40.7; 30.4; 27.8; 27.7. ESI-MS (*m*/*z*): [M+H]^+^ = 329.11. Anal. (C_18_H_24_N_4_O_2_) C, H, N Calcd: C 65.83%, H 7.37%, N 17.06%; Found: C 65.82%, H 7.36%, N 17.10%. m.p.: 69–70 °C.

*N*-(6-(pyrimidin-2-ylamino)hexyl)nicotinamide (**10**)

Compound **10** was prepared using 1,1-carbonyldiimidazole (0.081 g, 0.50 mmol), pyridine-3-carboxylic acid (0.062 g, 0.50 mmol), *N*^1^-(pyrimidin-2-yl)hexane-1,6-diamine (**1**) (0.097 g, 0.50 mmol), following the general procedure described above. Column chromatography: silica gel, CH_2_Cl_2_/MeOH 9:1, R_f_ = 0.45. After the chromatography the compound was purified by crystallisation in AcOEt. White solid, 0.071 g, 47% yield.

^1^H-NMR (400 MHz) (CD_3_CN) δ (ppm): 8.93 (d, 1H, *J* = 1.60 Hz, pyridine); 8.67 (dd, 1H, *J_1_* = 4.80 Hz, *J_2_* = 1.60 Hz, pyridine); 8.21 (d, 2H, *J* = 4.76 Hz, pyrimidine); 8.08 (dt, 1H, *J_1_* = 7.96 Hz, *J_2_* = 1.84 Hz, pyridine); 7.41 (ddd, 1H, *J_1_* = 7.92 Hz, *J_2_* = 4.80 Hz, *J_3_* = 0.76 Hz, pyridine); 7.11 (bs, 1H, -NH-C=O); 6.50 (t, 1H, *J* = 4.76 Hz, pyrimidine); 5.68 (bs, 1H, pyrimidine-NH-); 3.38–3.31 (m, 4H, -NH-CH_2_-CH_2_-CH_2_-CH_2_-CH_2_-CH_2_-NH-); 1.63–1.55 (m, 4H, -NH-CH_2_-CH_2_-CH_2_-CH_2_-CH_2_-CH_2_-NH-); 1.45–1.37 (m, 4H, -NH-CH_2_-CH_2_-CH_2_-CH_2_-CH_2_-CH_2_-NH-). ^13^C-NMR (100 MHz) (MeOD) δ (ppm): 167.7; 163.5; 159.2; 152.5; 149.0; 136.9; 132.2; 125.1; 111.0; 42.1; 41.0; 30.4; 30.3; 27.8; 27.7. ESI-MS (*m/z*): [M+H]^+^ = 299.93. Anal. (C_16_H_21_N_5_O) C, H, N Calcd: C 64.19%, H 7.07%, N 23.39%; Found: C 64.24%, H 7.08%, N 23.32%. m.p.: 99–101 °C.

*N*-(6-(pyrimidin-2-ylamino)hexyl)-1*H*-indole-2-carboxamide (**11**)

Compound **11** was prepared using 1,1-carbonyldiimidazole (0.081 g, 0.50 mmol), 1*H*-indole-2-carboxylic acid (0.081 g, 0.50 mmol), *N*^1^-(pyrimidin-2-yl)hexane-1,6-diamine (**1**) (0.097 g, 0.50 mmol), following the general procedure described above. The product was purified by crystallisation in CH_3_CN. White solid, 0.125 g, 74% yield.

^1^H-NMR (400 MHz) (*d_6_*-DMSO) δ (ppm): 11.51 (s, 1H, indole-NH-); 8.42 (bt, 1H, *J* = 5.44 Hz, -NH-C=O); 8.22 (d, 2H, *J* = 4.72 Hz, pyrimidine); 7.59 (d, 1H, *J* = 7.92 Hz, indole); 7.41 (d, 1H, *J* = 8.12 Hz, indole); 7.17–7.09 (m, 3H, 2H indole and pyrimidine-NH-); 7.02 (t, 1H, *J* = 7.12 Hz, indole); 6.50 (t, 1H, *J* = 4.72 Hz, pyrimidine); 3.25 (sextet, 4H, *J* = 6.72 Hz, -NH-CH_2_-CH_2_-CH_2_-CH_2_-CH_2_-CH_2_-NH-); 1.57–1.49 (m, 4H, -NH-CH_2_-CH_2_-CH_2_-CH_2_-CH_2_-CH_2_-NH-); 1.39–1.31 (m, 4H, -NH-CH_2_-CH_2_-CH_2_-CH_2_-CH_2_-CH_2_-NH-). ^13^C-NMR (100 MHz) (*d_6_*-DMSO) δ (ppm): 162.3; 161.0; 157.8; 136.3; 131.9; 127.1; 123.1; 121.4; 119.6; 112.3; 109.6; 102.2; 40.5; 38.7; 29.3; 28.9; 26.32; 26.28. ESI-MS (*m*/*z*): [M+H]^+^ = 338.01. Anal. (C_19_H_23_N_5_O) C, H, N Calcd: C 67.63%, H 6.87%, N 20.76%; Found: C 67.54%, H 6.89%, N 20.83%. m.p.: 187–189 °C.

*N*-(6-(pyridin-2-ylamino)hexyl)benzamide (**12**)

Compound **12** was prepared using 1,1-carbonyldiimidazole (0.065 g, 0.40 mmol), benzoic acid (0.049 g, 0.40 mmol), *N*^1^-(pyridin-2-yl)hexane-1,6-diamine (**2**) (0.077 g, 0.40 mmol), following the general procedure described above. Column chromatography: silica gel, CH_2_Cl_2_/isopropanol 9.2:0.8, R_f_ = 0.5. White solid, 0.095 g, 80% yield.

^1^H-NMR (400 MHz) (CD_3_CN) δ (ppm): 7.96 (dd, 1H, *J_1_* = 4.96 Hz, *J_2_* = 1.04 Hz, pyridine); 7.78–7.76 (m, 2H, aromatic); 7.53–7.49 (m, 1H, aromatic); 7.46–7.42 (m, 2H, aromatic); 7.38–7.34 (m, 1H, pyridine); 7.03 (bs, 1H, -NH-C=O); 6.48 (ddd, 1H, *J_1_* = 6.96 Hz, *J_2_* = 5.04 Hz, *J_3_* = 0.76 Hz, pyridine); 6.40 (d, 1H, *J* = 8.44 Hz, pyridine); 5.10 (bs, 1H, pyridine-NH-); 3.34 (q, 2H, *J* = 6.96 Hz, -CH_2_-NH-C=O); 3.22 (q, 2H, *J* = 6.88 Hz, pyridine-NH-CH_2_-); 1.62–1.54 (m, 4H, -NH-CH_2_-CH_2_-CH_2_-CH_2_-CH_2_-CH_2_-NH-); 1.46–1.35 (m, 4H, -NH-CH_2_-CH_2_-CH_2_-CH_2_-CH_2_-CH_2_-NH). ^13^C-NMR (100 MHz) (MeOD) δ (ppm): 170.2; 160.4; 147.8; 138.7; 135.9; 132.5; 129.5; 128.2; 112.9; 109.7; 42.6; 40.9; 30.5; 30.4; 27.9; 27.8. ESI-MS (*m*/*z*): [M+H]^+^ = 298.0. Anal. (C_18_H_23_N_3_O) C, H, N Calcd: C 72.70%, H 7.80%, N 14.13%; Found: C 72.72%, H 7.82%, N 14.10%. m.p.: 94–96 °C. 

2-phenyl-*N*-(6-(pyridine-2-ylamino)hexyl)acetamide (**13**)

Compound **13** was prepared using 1,1-carbonyldiimidazole (0.078 g, 0.48 mmol), phenylacetic acid (0.065 g, 0.48 mmol), *N*^1^-(pyridine-2-yl)hexane-1,6-diamine (**2**) (0.093 g, 0.48 mmol), following the general procedure described above. Column chromatography: silica gel, CH_2_Cl_2_/isopropanol 9:1, R_f_ = 0.6. White solid, 0.118 g, 79% yield.

^1^H-NMR (400 MHz) (*d_6_*-DMSO) δ (ppm): 8.04 (bt, 1H, *J* = 5.56 Hz, -NH-C=O); 7.92–7.90 (m, 1H, pyridine); 7.34–7.17 (m, 6H, 5H aromatic and 1H pyridine); 6.43–6.40 (m, 2H, pyridine); 6.36 (bt, 1H, *J* = 5.52 Hz, pyridine-NH-); 3.36 (s, 2H, Ar-CH_2_-C=O); 3.15 (t, 2H, *J* = 7.00 Hz, pyridine-NH-CH_2_-); 3.04–2.99 (m, 2H, -CH_2_-NH-C=O); 1.50–1.34 (m, 4H, -NH-CH_2_-CH_2_-CH_2_-CH_2_-CH_2_-CH_2_-NH-); 1.32–1.20 (m, 4H, -NH-CH_2_-CH_2_-CH_2_-CH_2_-CH_2_-CH_2_-NH-). ^13^C-NMR (100 MHz) (CDCl_3_) δ (ppm): 171.1; 158.5; 147.0; 138.1; 135.2; 129.5; 129.1; 127.4; 112.6; 107.0; 44.0; 42.1; 39.5; 29.4; 29.3; 26.6; 26.5. ESI-MS (*m*/*z*): [M+H]^+^ = 312.07. Anal. (C_19_H_25_N_3_O) C, H, N Calcd: C 73.28%, H 8.09%, N 13.49%; Found: C 73.31%, H 8.09%, N 13.45%. m.p.: 86–88 °C.

2-(4-phenoxyphenyl)-*N*-(6-(pyridin-2-ylamino)hexyl)acetamide (**14**)

Compound **14** was prepared using 1,1-carbonyldiimidazole (0.058 g, 0.36 mmol), (4-phenoxyphenyl)acetic acid (0.082 g, 0.36 mmol), *N*^1^-(pyridin-2-yl)hexane-1,6-diamine (**2**) (0.070 g, 0.36 mmol), following the general procedure described above. Column chromatography: silica gel, CH_2_Cl_2_/isopropanol/hexane 9:1:0.5, R_f_ = 0.44. White solid, 0.063 g, 43% yield.

^1^H-NMR (400 MHz) (CD_3_CN) δ (ppm): 7.96 (dd, 1H, *J*_1_ = 5.00 Hz, *J*_2_ = 1.08 Hz, pyridine); 7.38–7.34 (m, 3H, 2H aromatic and 1H pyridine); 7.27–7.24 (m, 2H, aromatic); 7.14–7.10 (m, 1H, aromatic); 7.00–6.97 (m, 2H, aromatic); 6.95–6.92 (m, 2H, aromatic); 6.49–6.46 (m, 2H, 1H pyridine and -NH-C=O); 6.40 (d, 1H, *J* = 8.44 Hz, pyridine); 5.09 (bs, 1H, pyridine-NH-); 3.40 (s, 2H, Ar-CH_2_-C=O); 3.22 (q, 2H, *J* = 6.92 Hz, pyridine-NH-CH_2_-); 3.12 (q, 2H, *J* = 6.76 Hz, -CH_2_-NH-C=O); 1.57–1.50 (m, 2H, -NH-CH_2_-CH_2_-); 1.48–1.25 (m, 6H, -NH-CH_2_-CH_2_-CH_2_-CH_2_-CH_2_-CH_2_-NH-). ^13^C-NMR (100 MHz) (MeOD) δ (ppm): 174.0; 160.4; 158.8; 157.6; 147.8; 138.7; 132.1; 131.5; 130.8; 124.3; 119.9; 119.7; 112.9; 109.7; 43.1; 42.5; 40.5; 30.33; 30.30; 27.73; 27.67. ESI-MS (*m*/*z*): [M+H]^+^ = 403.67. Anal. (C_25_H_29_N_3_O_2_) C, H, N Calcd: C 74.41%, H 7.24%, N 10.41%; Found: C 74.41%, H 7.25%, N 10.42%. m.p.: 97–99 °C.

*N*-(6-(pyridin-2-ylamino)hexyl)cinnamamide (**15**)

Compound **15** was prepared using 1,1-carbonyldiimidazole (0.058 g, 0.36 mmol), *trans*-cinnamic acid (0.053 g, 0.36 mmol), *N*^1^-(pyridin-2-yl)hexane-1,6-diamine (**2**) (0.070 g, 0.36 mmol), following the general procedure described above. Column chromatography: silica gel, CH_2_Cl_2_/isopropanol 9:1, R_f_ = 0.37. After the chromatography the compound was recrystallized from ethyl acetate with hexane. White solid, 0.049 g, 42% yield.

^1^H-NMR (400 MHz) (CD_3_CN) δ (ppm): 7.96 (dd, 1H, *J*_1_ = 5.04 Hz, *J*_2_ = 1.08 Hz, pyridine); 7.57–7.54 (m, 2H, aromatic); 7.48 (d, 1H, *J* = 15.76 Hz, Ar-CH=CH-); 7.42–7.34 (m, 4H, 3H aromatic and 1H pyridine); 6.64 (bs, 1H, -CH_2_-NH-C=O); 6.54 (d, 1H, *J* = 15.72 Hz, Ar-CH=CH-); 6.50–6.47 (m, 1H, pyridine); 6.42 (d, 1H, *J* = 8.44 Hz, pyridine); 5.17 (bs, 1H, pyridine-NH-); 3.28–3.22 (m, 4H, -NH-CH_2_-CH_2_-CH_2_-CH_2_-CH_2_-CH_2_-NH-); 1.61–1.49 (m, 4H, -NH-CH_2_-CH_2_-CH_2_-CH_2_-CH_2_-CH_2_-NH-); 1.45–1.36 (m, 4H, -NH-CH_2_-CH_2_-CH_2_-CH_2_-CH_2_-CH_2_-NH-). ^13^C-NMR (100 MHz) (MeOD) δ (ppm): 168.6; 159.5; 146.0; 141.5; 139.5; 136.3; 130.8; 129.9; 128.8; 121.9; 112.9; 110.5; 42.6; 40.5; 30.4; 30.2; 27.78; 27.75. ESI-MS (*m*/*z*): [M+H]^+^ = 324.10. Anal. (C_20_H_25_N_3_O) C, H, N Calcd: C 74.27%, H 7.79%, N 12.99%; Found: C 74.29%, H 7.77%, N 12.99%. m.p.: 92–95 °C.

#### 3.1.2. General Procedure for the Synthesis of Pyrimidine and Pyridine Carbamate Derivatives **16**–**19**

Intermediates **1** or **2** (1 eq) were dissolved in 10 mL of CHCl_3_ and TEA (1 eq) was added. Then a solution of the opportune chloroformate (1 eq) in 10 mL of CHCl_3_ was added slowly and dropwise. The reaction was stirred at room temperature for the indicated period of time (3–18 h). After this time, the mixture was washed with saturated aqueous solution of Na_2_CO_3_ (2 × 20 mL); the organic layer was dried over Na_2_SO_4_ and the solvent was evaporated under reduced pressure. The crude material was purified by column chromatography on silica gel.

Phenyl (6-(pyrimidin-2-ylamino)hexyl)carbamate (**16**)

Compound **16** was prepared following the general procedure described above; *N*^1^-(pyrimidin-2-yl)hexane-1,6-diamine (**1**) (0.101 g; 0.52 mmol), TEA (72 μL, d=0.726 g/mL, 0.52 mmol), phenyl chloroformate (65 μL, d = 1.248 g/mL, 0.52 mmol) were allowed to react for 18 h. Column chromatography: silica gel, AcOEt/isopropanol 9:1, R_f_ = 0.72. White solid, 0.125 g, 76% yield.

^1^H-NMR (400 MHz) (MeOD) δ (ppm): 8.24 (d, 2H, *J* = 4.84 Hz, pyrimidine); 7.35 (t, 2H, *J* = 7.72 Hz, aromatic); 7.19 (t, 1H, *J* = 7.40 Hz, aromatic); 7.08 (d, 2H, *J* = 7.72 Hz, aromatic); 6.56 (t, 1H, *J* = 4.84 Hz, pyrimidine); 3.36 (t, 2H, *J* = 7.04 Hz, pyrimidine-NH-CH_2_-); 3.18 (t, 2H, *J* = 6.92 Hz, -CH_2_-NH-C=O); 1.67–1.54 (m, 4H, -NH-CH_2_-CH_2_-CH_2_-CH_2_-CH_2_-CH_2_-NH-); 1.44–1.42 (m, 4H, -NH-CH_2_-CH_2_-CH_2_-CH_2_-CH_2_-CH_2_-NH-). ^13^C-NMR (50 MHz) (CDCl_3_) δ (ppm): 161.6; 157.9; 154.8; 151.2; 129.4; 125.3; 121.7; 110.3; 41.4; 41.2; 29.8; 29.5; 26.6; 26.5. ESI-MS (*m*/*z*): [M+H]^+^ = 314.99. Anal. (C_17_H_22_N_4_O_2_) C, H, N Calcd: C 64.95%, H 7.05%, N 17.82%; Found: C 64.89%, H 7.06%, N 17.85%. m.p.: 79–81 °C.

Benzyl (6-(pyrimidin-2-ylamino)hexyl)carbamate (**17**)

Compound **17** was prepared following the general procedure described above; *N*^1^-(pyrimidin-2-yl)hexane-1,6-diamine (**1**) (0.103 g; 0.53 mmol), TEA (74 μL, d = 0.726 g/mL, 0.53 mmol), benzyl chloroformate (75 μL, d = 1.212 g/mL, 0.53 mmol) were allowed to react for 4 h. Column chromatography: silica gel, AcOEt/MeOH/hexane 9.7:0.3:1.5, R_f_ = 0.62. White solid, 0.052 g, 30% yield.

^1^H-NMR (400 MHz) (*d_6_*-DMSO) δ (ppm): 8.27 (d, 1H, *J* = 4.80 Hz, pyrimidine); 7.37–7.21 (m, 7H, 5H aromatic, -NH-C=O, pyrimidine-NH-); 6.55 (t, 1H, *J* = 4.80 Hz, pyrimidine); 5.00 (s, 2H, Ar-CH_2_-O-C=O); 3.23 (bt, 2H, *J* = 6.08 Hz, pyrimidine-NH-CH_2_-); 2.97 (q, 2H, *J* = 6.60 Hz, -CH_2_-NH-C=O); 1.53–1.46 (m, 2H, -NH-CH_2_-CH_2_-); 1.42–1.36 (m, 2H, -NH-CH_2_-CH_2_-); 1.32–1.23 (m, 4H, -NH-CH_2_-CH_2_-CH_2_-CH_2_-CH_2_-CH_2_-NH-). ^13^C-NMR (100 MHz) (*d_6_*-DMSO) δ (ppm): 161.5; 157.8; 156.1; 137.3; 128.4; 127.75; 127.72; 109.6; 65.1; 40.6; 40.2; 29.4; 28.8; 26.2; 26.0. ESI-MS (*m*/*z*): [M+H]^+^ =328.96. Anal. (C_18_H_24_N_4_O_2_) C, H, N Calcd: C 65.83%, H 7.37%, N 17.06%; Found: C 65.86%, H 7.38%, N 17.01%. m.p.: 83–85 °C.

Phenyl (6-(pyridin-2-ylamino)hexyl)carbamate (**18**)

Compound **18** was prepared following the general procedure described above; *N*^1^-(pyridin-2-yl)hexane-1,6-diamine (**2**) (0.097 g; 0.50 mmol), TEA (70 μL, d = 0.726 g/mL, 0.50 mmol), phenyl chloroformate (63 μL, d = 1.248 g/mL, 0.50 mmol) were allowed to react for 3 h. Column chromatography: silica gel, CH_2_Cl_2_/MeOH 9.5:0.5, R_f_ = 0.62. White solid, 0.074 g, 47% yield.

^1^H-NMR (400 MHz) (MeOD) δ (ppm): 7.89 (d, 1H, *J* = 4.44 Hz, pyridine); 7.42–7.33 (m, 3H, 2H aromatic and 1H pyridine); 7.19 (t, 1H, *J* = 7.44 Hz, aromatic); 7.08 (d, 2H, 7.72 Hz, aromatic); 6.52–6.49 (m, 2H, pyridine); 3.26 (t, 2H, *J* = 7.04 Hz, pyridine-NH-CH_2_-); 3.18 (t, 2H, *J* = 6.96 Hz, -CH_2_-NH-C=O); 1.67–1.55 (m, 4H, -NH-CH_2_-CH_2_-CH_2_-CH_2_-CH_2_-CH_2_-NH-); 1.50–1.42 (m, 4H, -NH-CH_2_-CH_2_-CH_2_-CH_2_-CH_2_-CH_2_-NH-). ^13^C-NMR (50 MHz) (CDCl_3_) δ (ppm): 158.3; 154.8; 151.2; 146.4; 138.4; 129.4; 125.3; 121.7; 112.6; 107.1; 42.2; 41.2; 29.8; 29.4; 26.7; 26.5. ESI-MS (*m*/*z*): [M+H]^+^ = 314.00. Anal. (C_18_H_23_N_3_O_2_) C, H, N Calcd: C 68.98%, H 7.40%, N 13.41%; Found: C 69.00%, H 7.40%, N 13.40%. m.p.: 109–111 °C.

Benzyl (6-(pyridin-2-ylamino)hexyl)carbamate (**19**)

Compound **19** was prepared following the general procedure described above; *N*^1^-(pyridin-2-yl)hexane-1,6-diamine (**2**) (0.095 g; 0.49 mmol), TEA (68 μL, d = 0.726 g/mL, 0.49 mmol), benzyl chloroformate (69 μL, d = 1.212 g/mL, 0.49 mmol) were allowed to react for 18 h. Column chromatography: silica gel, AcOEt/isopropanol/hexane 9.8:0.2:2, R_f_ = 0.58. White solid, 0.052 g, 33% yield.

^1^H-NMR (400 MHz) (CD_3_CN) δ (ppm): 7.97 (d, 1H, *J* = 4.24 Hz, pyridine); 7.39–7.29 (m, 6H, 5H aromatic and 1H pyridine); 6.50–6.47 (m, 1H, pyridine); 6.40 (d, 1H, *J* = 8.44 Hz, pyridine); 5.61 (bs, 1H, -NH-C=O); 5.07 (bs, 1H, pyridine-NH-); 5.04 (s, 2H, Ar-CH_2_-O-C=O); 3.23 (q, 2H, *J* = 6.68 Hz, pyridine-NH-CH_2_-); 3.08 (q, 2H, *J* = 6.64 Hz, -CH_2_-NH-C=O); 1.59–1.52 (m, 2H, -NH-CH_2_-CH_2_-); 1.50–1.43 (m, 2H, -NH-CH_2_-CH_2_-); 1.41–1.27 (m, 4H, -NH-CH_2_-CH_2_-CH_2_-CH_2_-CH_2_-CH_2_-NH-). ^13^C-NMR (100 MHz) (MeOD) δ (ppm): 160.4; 158.9; 147.8; 138.7; 138.5; 129.4; 128.9; 128.7; 112.9; 109.7; 67.3; 42.6; 41.7; 30.9; 30.4; 27.8; 27.6. ESI-MS (*m*/*z*): [M+H]^+^ = 327.93. Anal. (C_19_H_25_N_3_O_2_) C, H, N Calcd: C 69.70%, H 7.70%, N 12.83%; Found: C 69.71%, H 7.71%, N 12.79%. m.p.: 64–66 °C.

### 3.2. Enzymatic Assays

*Electric eel* AChE (*Ee*AChE, EC 3.1.1.7), *equine* BChE (EC 3.1.1.8), acetylthiocholine iodide, 5,5′-dithio-bis-(2-nitrobenzoic acid) (DTNB), tacrine, donepezil and carbaryl, used as reference standard, were purchased from Sigma-Aldrich (Milan, Italy). All other chemical and biological reagents and solvents used were of the highest analytical, commercially available grade. The water, utilized for the preparation of the phosphate buffer and of the solutions of compounds, was distilled and filtered on nylon membrane filters with 0.2 μm pore size with the Millipore^®^ Filtration apparatus before each use. Micropipettes Labmate (BRAND Dig. 10–100 μL; Dig. 100–1000 μL; Dig. 0.1–2 μL) and Transferpette (HIGH TECH LAB LM200: 20–200 μL; LM5000: 1000–5000 μL) were used to collect the samples. The assays were carried out by double beam UV–Vis Lambda 40 Perkin Elmer spectrophotometer, using optical polystyrene cuvettes (10 × 10 × 45 mm, 340–800 nm optical transparency), each measure was repeated at least in triplicate. For data processing, UV-WIN Lab version 2.0, Perkin Elmer Corporation (Waltham, MA, USA) and SigmaPlot version 8.02 (Systat Software, Palo alto, CA, USA) were used. Vivaspin^®^ 2 Sartorius, with a membrane of 3000 MWCO (molecular weight cutoff) consisting of polyethersulfone (PES), cellulose triacetate (CTA) and hydrosart, was used for the ultrafiltration procedures. An ALC PK110 bench-top centrifuge was used for centrifugation. The spectrophotometric method of Ellman [33], with minor modifications, was used to evaluate the inhibition of cholinesterase enzymes. This method is based on the reaction of released thiocholine to give a coloured product, at a wavelength of 412 nm, with a chromogenic reagent DTNB. The absorbance was recorded at 412 nm between 0 and 1.6 min and the absorbance variation, utilized for the kinetic assay, and was measured between 0.5 and 1.5 min to allow the stabilisation of the UV–Vis lamp and of the solution. The method is extremely sensitive to variations in the order of microliters: the standard deviations (less than 5%) of the values obtained are compatible with the experimental errors associated with the use of micropipettes. Each compound tested was dissolved in the opportune quantity of DMSO in order to obtain a final cuvette DMSO content < 0.033%, which does not affect the enzyme activity. *Ee*AChE and *eq*BChE were periodically tested to evaluate the effective enzymatic activity.

#### 3.2.1. Percent Inhibition of *Ee*AChE and *eq*BChE

For all the synthesized compounds, the percentages of inhibition towards AChE of *Electrophorus electricus* (*Ee*AChE) and equine BChE (*eq*BChE) were evaluated.

An amount of 3.0 mL of a solution in 0.1 M phosphate buffer (pH = 7.4) containing DTNB (0.25 mM) and *Ee*AChE (0.083 UmL^−1^) or *eq*BChE (0.083 UmL^−1^) were placed in a polystyrene cuvette of 1.0 cm path length; 1 µL of a solution in DMSO of the tested compound was added to obtain in cuvette concentration range 9 µM–0.09 µM. With this solution the blank was made. To start the reaction, 30 µL of a solution in 0.1 M phosphate buffer (pH = 7.4) of acetylthiocholine (10 mM) were added in order to obtain a final concentration of acetylthiocholine equal to 100 µM. The increase in the absorbance, due to the production of the yellow 2-nitro-5-thiobenzoic anion, was recorded at 412 nm at 25 °C, and the absorbance variation was measured between 0.5 and 1.5 min. As a control, an identical solution of the enzyme without the inhibitor was processed following the same protocol to determine the 100% of enzyme activity.

Each experiment was repeated at least in triplicate. The potency of each compound to inhibit *Ee*AChE or *eq*BChE activity was expressed as percent inhibition calculated using the following equation:

$$Inhibition\;\left(\%\right)=\frac{A_{c}-A_{i}}{A_{c}}\times 100$$

where *A_i_* and *A_c_* represent the average of absorbance variation in presence of inhibitor and without the inhibitor, respectively.

#### 3.2.2. Time Dependent Inhibition Assay for Carbamate Derivatives **16**–**19**

For carbamate derivatives **16**–**19,** the percentages of inhibition were measured both at zero time, according to the procedure described above, and after incubation of 1 h at 25 °C. In this case, for each compound, three cuvettes were prepared with 3.0 mL of a solution in 0.1 M phosphate buffer (pH = 7.4) containing DTNB (0.25 mM) and *Ee*AChE (0.083 UmL^−1^) or *eq*BChE (0.083 UmL^−1^) and with 1 μL of inhibitor solution in DMSO, in order to obtain final compound concentrations equal to 9 μM for *Ee*AChE and equal to 900 nM for *eq*BChE; in parallel, three control cuvettes were prepared with 3.0 mL of a solution in 0.1 M phosphate buffer containing DTNB (0.25 mM) and enzyme (0.0833 UmL^−1^), without the inhibitor, to determine the 100% of enzyme activity. The cuvettes were prepared consecutively at intervals of 3 min to allow equal incubation times. Each cuvette was incubated for 1 h at 25 °C. Then the blank was made, and the reaction was activated by adding 30 μL of a solution in 0.1 M phosphate buffer (pH = 7.4) of acetylthiocholine (10 mM), in order to obtain a final concentration of substrate equal to 100 μM. The absorbance variation was measured at 412 nm at 25 °C, between 0.5 and 1.5 min, and the percentage of inhibition after incubation was calculated.

For carbamate **18,** the percentages of inhibition toward *eq*BChE were determined at increasing incubation times. The same protocol described above was followed, but with different incubation times, every 15 min for 2 h. For each incubation time three cuvettes were prepared as previously described, with a final compound concentration equal to 700 nM.

#### 3.2.3. Determination of Constant and Mechanism of Inhibition vs. *eq*BChE

The constant and the mechanism of inhibition vs. *eq*BChE were determined for the compounds **12** and **14**. For each compound a 500 μM stock solution was prepared in H_2_O/DMSO and diluted in water to prepare solutions of opportune concentrations (10, 30 and 60 μM) in order to introduce the cuvette volumes ranging from 60 to 100 μL to obtain the desired final concentrations (200–1800 nM). The maximum amount of DMSO used for the preparation of the stock solution was calculated in order to obtain a DMSO content in cuvette of <0.033%.

An amount of 3.0 mL of 0.1 M phosphate buffer (pH = 7.4), containing DTNB (0.25 mM) and *eq*BChE (0.083 UmL^−1^), were mixed with the opportune volume of inhibitor, to obtain, in the cuvette, a final inhibitor concentration between 200 and 1800 nM. With this solution the blank was made. The reaction was started by adding the proper volume of a solution in 0.1 M phosphate buffer (pH = 7.4) of acetylthiocholine (10 mM) to the enzyme–inhibitor mixture, in order to obtain a final concentration of substrate equal to 100–300 µM. Each determination was repeated for five times. The absorbance variations were measured at 412 nm at 25 °C between 0.5 and 1.5 min. From these data, the values of the enzymatic hydrolysis rate, expressed as μmol of substrate hydrolysed in a minute by an enzymatic unit, were obtained.

In order to determine the inhibition constants (K_i_) and inhibition mechanism the rates of hydrolysis at three different concentrations of substrate in the presence of five different concentration of inhibitor were measured. The recorded data were analyzed by the enzyme kinetic module of SigmaPlot (version 8.02), plotting the reciprocal of rate of hydrolysis (1/v, min/μM) vs. the concentration of inhibitor (nM), according to Dixon’s method [34] in order to find the best fitting model of inhibition, as indicated by calculated linear regression coefficient R^2^.

#### 3.2.4. Determination of IC_50_ vs. *eq*BChE for Compound **18**

For compound **18** the IC_50_ value vs. *eq*BChE was calculated. Eight different inhibitor solutions in DMSO were prepared, having suitable concentrations, so as to add 1 μL of these solutions to the assay mixture to obtain a final concentration between 50 and 1500 nM, with a final DMSO content <0.033%. For each inhibitor concentration the same protocol described above for the determination of the percentage of inhibition towards *eq*BChE (0.0833 UmL^−1^) after incubation for 1 h at 25 °C and then activating the reaction with acetylthiocholine (100 μM) was followed. The recorded data were analyzed by the enzyme kinetic module of SigmaPlot, plotting the percentages of inhibition as a function of the different concentrations of inhibitor, expressed in logarithmic scale, in order to obtain the sigmoid graph, from which it was possible to determinate the IC_50_ value of the tested compound. The IC_50_ value was confirmed by repeating the experiment twice.

#### 3.2.5. Study of Reversibility of Inhibition of Compound **18** vs. *eq*BChE

Three solutions were prepared in 0.1 M phosphate buffer (pH = 7.4), containing *eq*BChE (0.50 UmL^−1^), DTNB (0.3 mM) and acetylthiocholine (10 mM), respectively. A solution of compound **18** in DMSO (2.7 mM) was prepared. Then six Vivaspin^®^ were used:

- three were prepared with 2.5 mL of *eq*BChE solution (0.50 UmL^−1^) and 5 µL of inhibitor solution;
- three were prepared with 2.5 mL of *eq*BChE solution (0.50 UmL^−1^) and 5 µL of DMSO.

The Vivaspin^®^ thus prepared were incubated for 1 h at 25 °C. After this time the percentage of inhibition was determined following the Ellman method. The blank was recorded by taking 500 µL from the Vivaspin^®^ and diluting them in the cuvette in 2.5 mL of DTNB solution (0.3 mM). The reaction was activated by adding 30 μL of acetylthiocholine solution (10 mM), so as to obtain a final concentration of 100 µM in the cuvette. The absorbance variation was measured between 0.5 and 1.5 min at 412 nm, repeating the measurement for each of the six Vivaspin^®^. 

After this procedure the first centrifugation of the Vivaspin^®^ was carried out for 60 min at 4000 rpm and subsequently the solutions that crossed the membrane and settled in the filtrate tube were removed. In this way the solutions present in the concentrator body were concentrated up to 100 µL. To these solutions 1 mL of 0.1 M phosphate buffer (pH = 7.4) was added and the second centrifugation was carried out for 30 min at 4000 rpm. The solutions present in the concentrator body were concentrated up to 200 µL. The second washing was carried out, adding 1 mL of buffer and centrifuging for 30 min at 4000 rpm. The solutions present in the concentrator body were concentrated up to 250 µL. A third wash was carried out adding 1 mL of buffer and centrifuging for 50 min at 4000 rpm. The solutions present in the concentrator body were concentrated up to 150 µL. At this point, to recover the concentrated solution, the filtrate tube was removed, the concentrator body was turned upside down and inserted inside the concentrate recovery cap. The Vivaspin^®^ was centrifuged for 10 min at 4000 rpm. After this time the recovered solution was taken up with 3 mL of DTNB solution (0.3 mM) and transferred into the cuvette: blank was recorded. The reaction was activated when 30 µL of substrate solution (10 mM) were added so as to have a final concentration of 100 µM in the cuvette. The absorbance variation between 0.5 and 1.5 min was measured at 412 nm, repeating the measurement for each of the recovered solutions. With these data the percentage of inhibition was calculated using the following equation:

$$Inhibition\;\left(\%\right)=\frac{A_{rc}-A_{ri}}{A_{rc}}$$

where *A_ri_* and *A_rc_* represent the average of absorbance variation in the solutions recovered from the Vivaspin^®^, initially prepared with the inhibitor, and in the solutions recovered from the Vivaspin^®^, initially prepared without the inhibitor, respectively.

As a positive control carbaryl was used, for which the same procedure was followed, using a 27 mM carbaryl stock solution in DMSO.

### 3.3. Molecular Docking Studies and ADME Prediction

All in silico simulations were carried out by means of Schrödinger Suite 2018-1 [45].

The newest X-ray crystallographic structures of both *h*AChE and *h*BChE in complex with a triphenylphosphonium conjugate with the 6ZWE and 6ZWI PDB codes, respectively, were adopted for the computational studies [37]. *h*ChEs structures were prepared by using the Maestro Protein Preparation Wizard [46] tool and were refined to optimize hydrogen-bonds and energy minimized by using OPLS_2005 as force field at pH 7.4 [47,48].

The homology between the human and non-human ChEs isoforms used for the enzymatic assays was assessed by the sequence alignment performed by Prime [49]. 

All synthesized ligands were submitted to 5000 steps of Monte Carlo conformational search applied to all rotatable bonds. The water solvent effect was considered using the implicit model GB/SA [50]. The global minimum energy structures were used to carry out molecular docking simulations. Then, target binding sites were defined by means of a regular grid box of about 27,000 Å^3^ centred on the catalytic serine residues. All docking simulations were computed using Glide [51] ligand flexible algorithm at standard-precision (SP) level [52].

The best docked poses of the most interesting inhibitors resulted through the enzymatic assays of pyridine amides and carbamate derivatives were submitted to 300 ns of molecular dynamics (MDs) simulations by using Desmond ver. 4.2 [53]. The system was solvated in TIP3P explicit solvent model and counter ions were added to neutralize the system net charge. After the optimisation of the solvated model, we relaxed the system with Martyna-Tobias_Klein isobaric-isothermal ensemble (MTK_NPT). This preliminary stage included two energy minimisations of 2000 steps: in the first run, the system was restrained with a force constant of 50 kcal·mol^−1^·A^−1^, while in the second one all the system was released without any restrains. The following conditions for MDs were used: NPT ensemble, a temperature of 300 K, a pressure of 1 bar with the Berendsen thermostat-barostat, a recording interval equal to 250 ps both for energy and for trajectory collecting 1000 frames for each simulation [54].

ADME descriptors of the most active compounds were predicted using the SwissADME public server [43]. The following ADME parameters were selected: Molecular weight (MW); number H-bond acceptors (HBA); number H-bond donors (HBD); number of heavy atoms (heavy atoms); number rotatable bonds (RB); topological polar surface area in Å^2^ (TPSA); Octanol/water partition coefficient (MLogP); water solubility (LogS ESOL); water solubility class (Sol class); gastrointestinal absorption (GI); number of Lipinski’s rule of five violations (Lipinski viol) [44].

### 3.4. Chelation Studies

FeCl_3_·6H_2_O, CuSO_4_·5H_2_O, Zn(NO_3_)_2_·6H_2_O and methanol, used for chelation studies, were purchased from Sigma-Aldrich. Micropipettes Labmate (BRAND Dig. 10–100 μL; Dig. 100–1000 μL) and Transferpette (HIGH TECH LAB LM200: 20–200 μL; LM5000: 1000–5000 μL) were used to collect the samples. The assays were carried out by double beam UV–Vis Lambda 40 Perkin Elmer spectrophotometer, using quartz cuvettes (Optech, type S/Q/10). Data were elaborated using UV-WIN Lab Version 2.0, Perkin Elmer Corporation, Microsoft Excel 2010 and Spekwin32.

#### 3.4.1. UV-Vis Titration

For the UV–Vis titration, solutions 10^−3^ M in MeOH of compounds **12**, **14** and **18** were prepared. Stock solutions 1 M of CuSO_4_·5H_2_O and Zn(NO_3_)_2_·6H_2_O in water and 0.1 M of FeCl_3_·6H_2_O in MeOH were prepared and diluted with MeOH to obtain different solutions with suitable concentrations, so as to add 2.85 mL of these solutions to 150 µL of ligand solution, to obtain in 3 mL of assay mixture a final molar ratio of metal ion to ligand between 0.1 and 20. UV–Vis spectra were recorded from 210 to 450 nm.

For the first measure, 150 µL of ligand solution were diluted in 2.85 mL of MeOH into sample cuvette, while only MeOH was placed in reference cuvette and the UV–Vis spectrum was recorded. For subsequent measurements, 2.85 mL of the opportune metal solution were placed both in a sample cuvette and in a reference cuvette; then 150 µL of ligand solution were added to a sample cuvette, while 150 µL of MeOH were added to the reference cuvette and the UV–Vis spectra were recorded. The spectra thus obtained were superimposed to observe the variations present between the ligand alone and in presence of increasing amount of metal.

#### 3.4.2. Job’s Plot

To determine the stoichiometric coefficients of the complexes, Job’s method [41] was used, which requires the mixing, in appropriate proportions, of the equimolar solution of metal ion and ligand, so that the final volume and the total moles present in the cuvette are equal for each measurement. The absorbance values were recorded at the wavelengths, extrapolated from the UV–Vis titration spectra, where the maximum variation of absorbance was observed. At the same time two solutions in MeOH of equal concentration were prepared, one of ligand and one of metal ion, diluting the solutions 1 M of CuSO_4_·5H_2_O and Zn(NO_3_)_2_·6H_2_O and 0.1 M of FeCl_3_·6H_2_O previously prepared. The concentrations of the solutions used and the wavelengths in which the absorbance was recorded were: 2.96·10^−4^ M for **12** and Fe^3+^–251, 320 nm; 2.83·10^−4^ M for **14** and Fe^3+^–250, 310 nm; 2.90·10^−4^ M for **18** and Fe^3+^–245, 310 nm; 6.00·10^−4^ M for **18** and Cu^2+^–330 nm.

For each determination 9–20 measurements were made, introducing the appropriate volumes of metal and ligand solutions in the sample cuvette to obtain mole fractions of the ligand in the range 0.1–0.9, while appropriate volumes of ligand solution and MeOH were placed in the reference cuvette, to obtain the same concentration of ligand in the sample cuvette. The absorbance values of the metal ion, calculated by the Lambert–Beer relation, known the extinction coefficients ε of the metal ion at used wavelengths and the nominal concentrations of the metal ion for each measure, were algebraically subtracted from the recorded absorbance values, in order to obtain the exclusive absorbance variations due to the complex formation. 

The resulting ΔA were reported in graph as a function of the mole fraction of the ligand and through the intersections of the linear regression lines, the mole fraction X, which caused the maximum variation in absorbance, was determined and used to calculate the value of the coefficient n, which corresponds to the number of ligand molecules per cation, applying the following equation:

$$n=\frac{X}{1-X}$$

### 3.5. Inhibition of Amyloid and Tau Aggregation

#### 3.5.1. Cloning and Overexpression of the Aβ_42_ Peptide

*E. coli* competent cells BL21 (DE3) were transformed with the pET28a vector (Novagen, Inc., Madison, WI, USA) carrying the DNA sequence of Aβ_42_. Because of the addition of the initiation codon ATG in front of both genes, the overexpressed peptide contains an additional methionine residue at its N terminus. For overnight culture preparation, an amount of 10 mL of the M9 minimal medium containing 50 μg·mL^−1^ of kanamycin was inoculated with a colony of BL21 (DE3) bearing the plasmid to be expressed at 37 °C. For expression of the Aβ_42_ peptide, the required volume of overnight culture to obtain 1:500 dilution was added into the fresh M9 minimal medium containing 50 μg·mL^−1^ of kanamycin and 250 μM Th-S. The bacterial culture was grown at 37 °C and 250 rpm.

When the cell density reached OD_600_ = 0.6, an amount of 980 μL of culture was transferred into Eppendorf tubes of 1.5 mL with 10 μL of each compound to be tested in DMSO and 10 μL of isopropyl 1-thio-β-d-galactopyranoside (IPTG) at 100 mM. The final concentration of the drug was fixed at 100 μM. The samples were grown overnight at 37 °C and 1400 rpm using a ThermoMixer (Eppendorf, Hamburg, Germany). As a negative control (maximal amyloid presence), the same amount of DMSO without the drug was added in the sample. In parallel, non-induced samples (in the absence of IPTG) were also prepared and used as positive controls (non-amyloid presence). In addition, these samples were used to assess the potential intrinsic toxicity of the compounds and to confirm the correct bacterial growth.

#### 3.5.2. Cloning and Overexpression of the Tau Protein

*E. coli* BL21 (DE3) competent cells were transformed with pTARA containing the RNA-polymerase gene of T7 phage (T7RP), under the control of the promoter PBAD. *E. coli* BL21 (DE3), with pTARA competent cells, were transformed with the pRKT42 vector encoding four repeats of the tau protein in two inserts. For overnight culture preparation, 10 mL of the M9 medium containing 0.5% of glucose, 50 μg·mL^−1^ of ampicillin, and 12.5 μg·mL^−1^ of chloramphenicol was inoculated with a colony of BL21 (DE3) bearing the plasmids to be expressed at 37 °C. For the expression of the tau protein, the required volume of overnight culture to obtain 1:500 dilution was added to the fresh M9 minimal medium containing 0.5% of glucose, 50 μg·mL^−1^ of ampicillin, 12.5 μg·mL^−1^ of chloramphenicol, and 250 μM Th-S. The bacterial culture was grown at 37 °C and 250 rpm. When the cell density reached OD_600_ = 0.6, an amount of 980 μL of culture was transferred into Eppendorf tubes of 1.5 mL with 10 μL of each compound to be tested in DMSO and 10 μL of arabinose at 25%. The final concentration of the drug was fixed at 100 μM. The samples were grown overnight at 37 °C and 1400 rpm using a ThermoMixer (Eppendorf, Hamburg, Germany). As a negative control (maximal presence of tau), the same amount of DMSO without the drug was added in the sample. In parallel, non-induced samples (in the absence of arabinose) were also prepared and used as positive controls (absence of tau). In addition, these samples were used to assess the potential intrinsic toxicity of the compounds and to confirm the correct bacterial growth.

#### 3.5.3. Thioflavin S (Th-S) Steady-State Fluorescence

Th-S (T1892) and other chemical reagents were purchased from Sigma (St. Louis, MO, USA). The Th-S stock solution (2500 mM) was prepared in double-distilled water purified through a Milli-Q system (Millipore, Burlington, MA, USA). Th-S fluorescence and absorbance were tracked using a DTX 800 plate reader Multimode Detector equipped with a Multimode Analysis Software (Beckman-Coulter, Indianapolis, IN, USA). Filters of 430/35 and 485/20 nm were used for the excitation and emission wavelengths, respectively. A filter of 535/25 nm was also used for the absorbance determination. To normalize the Th-S fluorescence as a function of the bacterial concentration, OD_600_ was obtained using a Shimadzu UV-2401 PC UV–Vis spectrophotometer (Shimadzu, Japan). Note that the fluorescence normalisation was carried out considering 100% of the Th-S fluorescence of the bacterial cells expressing the peptide or protein in the absence of the drug and 0% of the Th-S fluorescence of the bacterial cells not expressing the peptide or protein. A minimum of five independent assays (with three replicates for assay) was performed for each tested compound. More assays were performed to obtain a SEM <5% with a maximum of 10 independent assays.

### 3.6. Cytotoxicity Assay

The compounds **12**, **14** and **18** were evaluated for cell viability effects using the MTT assay. Briefly, U-87 MG Cell Line from human brain (glioblastoma astrocytoma) (ATCC, Manassas, VA, USA) were seeded into 96-well microtiter plates (NunclonTM, Nunc, Germany) at a density of 1.5 × 10^4^ cells/well. After 24 h cells were exposed to increased concentrations of tested compounds (1, 5, 10 and 50 µM) in cell culture medium. After an incubation time of 24 h, the medium was replaced by a new, fresh medium containing 0.5 mg/mL MTT (Sigma, Deisenhofen, Germany). After 2 h at 37 °C in 5%, CO_2_ unreacted dye was removed and the cells were dissolved by DMSO (Merck, Darmstadt, Germany). Absorbance was read at 570 nm by a microtiter spectrophotometer plate reader. The data were expressed as absorbance relative to untreated cells in the same experiment and standardized to 100%. All data points were performed in triplicate and at least three independent experiments.

## 4. Conclusions

Based on the interesting results previously reported for some pyrimidine and pyridine diamine derivatives [32], new compounds, containing a 2-amino-pyrimidine or a 2-amino-pyridine moiety connected to an aromatic group by a flexible alkyl chain and an amide or carbamic group, were designed and tested both in vitro and in silico to evaluate their ChEs inhibitory ability and to propose possible binding mode with these enzymes. Regarding the synthesized compounds, the determination of the percentages of inhibition toward *Ee*AChE and *eq*BChE were carried out at concentrations equal to 9−0.9 μM. In general, among amide derivatives, the pyrimidines are weak inhibitor of both ChEs, while the pyridines appear more potent inhibitors than the pyrimidine analogues, in particular toward *eq*BChE. Additionally, among carbamates derivatives, pyridine carbamates show greater inhibitory potency on both ChEs compared to the corresponding pyrimidine derivatives. Moreover, the carbamate derivatives are more selective towards *eq*BChE than *Ee*AChE. The increase in the percentages of inhibition towards both enzymes over time, in particular for the phenylcarbamates **16** and **18**, suggests that these compounds could act as carbamoylating agents. If compared with the corresponding amine derivatives already studied, the replacement of the amino group with the amide (pyridine **12**) or the carbamate (pyrimidine **16** and pyridine **18**) reduce the inhibitory activity on *Ee*AChE, but enhance it toward *eq*BChE, leading to selective *eq*BChE inhibitors. Among the studied compounds, the most potent *eq*BChE inhibitors are the pyridine amides **12** (K_i_ = 2.988 ± 0.190 µM) and **14** (K_i_ = 0.621 ± 0.043 µM), with an unsubstituted phenyl ring or a 4-phenoxybenzyl ring on one side of the aliphatic chain, respectively, and the pyridine phenylcarbamate **18** (IC_50_ = 0.454 ± 0.082 µM).

In silico analyses allowed to identify and rationalize the crucial pharmacophore features for BChE inhibition, which are represented by the pyridine moiety and the alkyl chain of six methylene units. Additionally, the presence of phenylcarbamate in compound **18** resulted in enhancing the binding affinity, owing to the long-lasting interaction with Ser287, observed by MDs run. This interaction seems to maximize the selectivity toward BChE enzyme.

As already evidenced for the pyrimidine and pyridine amine derivatives previously studied, metal chelation analyses confirmed that the tested compounds **12**, **14** and **18** have the ability to chelate Fe^3+^ and Cu^2+^ ions, due to the presence of two adjacent nitrogen atoms in the 2-amino-pyridine moiety.

In cellulo studies on *E. coli* cells showed that the tested compounds have a weak anti-aggregating activity toward both Aβ_42_ and tau proteins, while cytotoxicity assays revealed a low toxicity on human brain cells.

In conclusion, among the molecules studied in this work, compounds **12**, **14** and **18** are selective inhibitors of *eq*BChE and retain the chelating ability, the low toxicity and the good predicted ADME parameters already shown for amine analogues.

## Figures and Tables

**Table 1:** Inhibition of *Ee*AChE and *eq*BChE activities by pyrimidine and pyridine amide derivatives **3**–**15**.

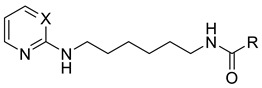			% Inhibition vs. *Ee*AChE ± SD ^a^		% Inhibition vs. *eq*BChE ± SD ^a^	
Cmp	R	X	[I] 9 µM	[I] 900 nM	[I] 9 µM	[I] 900 nM
**3**	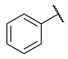	N	2.5 ± 4.1	nd ^b^	8.5 ± 1.7	nd ^b^
**4**	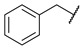	N	5.0 ± 4.4	nd ^b^	5.1 ± 4.7	nd ^b^
**5**	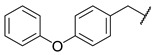	N	10.8 ± 2.7	nd ^b^	16.6 ± 5.5	nd ^b^
**6**	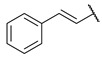	N	ns ^c^	7.0 ± 1.6	ns ^c^	na ^d^
**7**	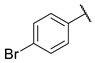	N	11.2 ± 5.0	nd ^b^	7.4 ± 2.0	nd ^b^
**8**	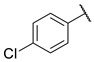	N	9.9 ± 0.8	nd ^b^	2.1 ± 3.5	nd ^b^
**9**	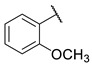	N	7.2 ± 0.8	Nd ^b^	9.3 ± 2.4	nd ^b^
**10**	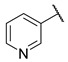	N	na ^d^	nd ^b^	na ^d^	nd ^b^
**11**	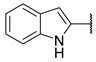	N	ns ^c^	5.5 ± 1.4	ns ^c^	14.1 ± 4.7
**12**	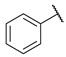	CH	34.0 ± 2.1	nd ^b^	80.5 ± 1.1	22.3 ± 1.6
**13**	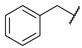	CH	46.7 ± 1.1	nd ^b^	49.1 ± 3.2	4.3 ± 5.6
**14**	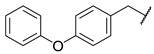	CH	42.6 ± 1.4	nd ^b^	87.6 ± 1.5	45.2 ± 2.3
**15**	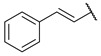	CH	45.4 ± 2.5	nd ^b^	64.7 ± 2.9	31.5 ± 3.1
**Tacrine**			100	97.6 ± 0.1	100	99.7 ± 0.3

^a^ Data are the average of three replicates; ^b^ nd stands for not determined; ^c^ ns stands for not soluble; ^d^ na stands for not active.

**Table 2:** Inhibition of *Ee*AChE and *eq*BChE activities by pyrimidine and pyridine carbamate derivatives **16–19**.

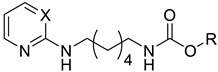			% Inhibition vs. *Ee*AChE ± SD ^a^		% Inhibition vs. *eq*BChE ± SD ^a^		
Cmp	R	X	[I] = 9 µM		[I] = 9 µM	[I] = 900 nM	
			t = 0	t = 1 h	t = 0	t = 0	t = 1 h
**16**	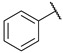	N	na ^b^	7.2 ± 0.8	38.9 ± 2.5	6.4 ± 6.8	66.4 ± 5.9
**17**	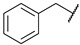	N	na ^b^	10.9 ± 1.4	16.0 ± 3.7	na ^b^	13.3 ± 3.9
**18**	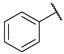	CH	29.4 ± 0.7	41.6 ± 0.7	85.8 ± 1.0	35.2 ± 0.3	79.6 ± 5.3
**19**	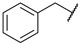	CH	8.4 ± 3.4	45.4 ± 2.7	80.7 ± 2.3	25.9 ± 2.9	32.4 ± 7.5

^a^ Data are the average of three replicates; ^b^ na stands for not active.

**Table 3:** Inhibition constants (K_i_) and inhibition mechanism of compounds **12** and **14** on *eq*BChE.

	*eq*BChE		
Cmp	Mechanism	K_i_ ± SD (µM)	R^2^
**12**	Competitive	2.988 ± 0.190	0.987
**14**	Competitive	0.621 ± 0.043	0.980

**Table 4:** IC_50_ on *eq*BChE for compound **18** and for tacrine and donepezil, used as the reference standards.

	*eq*BChE
Cmp	IC_50_ ± SE (nM)
**18**	454.3 ± 82.4
**Tacrine**	3.7 ± 0.5
**Donepezil**	1727 ± 200

**Table 5:** Job’s plot data for the tested compounds (**12**, **14**, **18**) in presence of Fe^3+^ and Cu^2+^. The table shows the wavelength values (λ) in which the absorbance variation was measured, the molar ratios (X) that cause the maximum variation of absorbance and the coordination values (n) which correspond to the number of ligand molecules per cation.

	Fe^3+^			Cu^2+^		
Cmp	λ (nm)	X	n	λ (nm)	X	n
**12**	251	0.69	2	-	-	-
**14**	250	0.70	2	-	-	-
**18**	245	0.69	2	330	0.49	1

**Table 6:** Inhibition of Aβ_42_ aggregation and tau aggregation by tested compounds.

	Aβ_42_ Aggregation		Tau Aggregation	
Cmp	% Inhibition [I] = 100 µM	SEM ^a^	% Inhibition [I] = 100 µM	SEM ^a^
**12**	11.9	4.5	11.0	6.9
**14**	13.3	6.2	10.9	6.4
**18**	23.1	3.4	15.7	2.0
**Ref ^b^**	98.8	1.0	94.7	3.1

^a^ A minimum of five independent assays (with three replicates for assay) was performed for each tested compound. More assays were performed to obtain a SEM < 5% with a maximum of 10 independent assays. ^b^ The known antiamyloid drug, DP-128 (100 μM), was used as a reference compound.

**Table 7:** Physicochemical properties and ADME parameters: Molecular Weight (MW); number H-bond acceptors (HBA); number H-bond donors (HBD); number of heavy atoms (heavy atoms); number rotatable bonds (RB); topological polar surface area in Å^2^ (TPSA); octanol/water partition coefficient (MLogP); water solubility (LogS ESOL); water solubility class (Sol class); gastrointestinal absorption (GI); blood–brain barrier permeation (BBB); number of Lipinski’s rule of five violations (Lipinski viol).

Cmp	MW	HBA	HBD	Heavy Atoms	RB	TPSA	MLogP	LogS ESOL	Sol Class	GI	BBB	Lipinski Viol
**12**	297.39	2	2	22	10	54.02	2.54	−3.68	Soluble	High	Yes	0
**14**	403.52	3	2	30	12	63.25	3.29	−5.10	Moderately soluble	High	Yes	0
**18**	313.39	3	2	23	11	63.25	2.53	−3.88	Soluble	High	Yes	0

## Data Availability

Data is contained within the article and Supplementary Materials.

## Supplementary Materials

The following supporting information can be downloaded at: https://www.mdpi.com/article/10.3390/ph15060673/s1. Dixon’s plots of compounds **12** and **14** towards *eq*BChE (Figures S1 and S2); IC_50_ graph of compound **18** towards *eq*BChE (Figure S3); In silico studies (Figures S4–S7, Tables S1 and S2); UV–Vis titration spectra and Job’s plots for selected compounds (Figures S8–S17).
