# Supplementary material for: Design, Synthesis, and In Vitro, In Silico and In Cellulo Evaluation of New Pyrimidine and Pyridine Amide and Carbamate Derivatives as Multi-Functional Cholinesterase Inhibitors
Source: Pharmaceuticals (Basel). 2022 May 27;15(6):673. doi: 10.3390/ph15060673 (PMC9227096; doi:10.3390/ph15060673)
Supplement: Supplementary file 1 [file pharmaceuticals-15-00673-s001.zip › pharmaceuticals-1723520-supplementary.pdf]

Design, Synthesis, and In Vitro, In Silico and In Cellulo Evaluation of New Pyrimidine and Pyridine Amide and Carbamate Derivatives as Multi-Functional Cholinesterase Inhibitors

Martina Bortolami [1], Fabiana Pandolfi [1], Valeria Tudino [2], Antonella Messore [2], Valentina Noemi Madia [2], Daniela De Vita [3], Roberto Di Santo [2,4], Roberta Costi [2,4], Isabella Romeo [5,6], Stefano Alcaro [5,6,*], Marisa Colone [7],
Annarita Stringaro [7], Alba Espargaró [8,9], Raimon Sabatè [8,9] and Luigi Scipione [2,*]

1 Department of Scienze di Base e Applicate per l'Ingegneria, Sapienza University of Rome, Via Castro Laurenziano 7, 00185 Rome, Italy; martina.bortolami@uniroma1.it (M.B.); fabiana.pandolfi@uniroma1.it (F.P.)
2 Department of Chimica e Tecnologia del Farmaco, Sapienza University of Rome, Piazzale Aldo Moro 5, 00185 Rome, Italy; valeria.tudino@uniroma1.it (V.T.); antonella.messore@uniroma1.it (A.M.); valentinanoemi.madia@uniroma1.it (V.N.M.)
3 Department of Environmental Biology, Sapienza University of Rome, Piazzale Aldo Moro 5, 00185 Rome, Italy; daniela.devita@uniroma1.it
4 Instituto Pasteur, Fondazione Cenci Bolognetti, Sapienza University of Rome, Piazzale Aldo Moro 5, 00185 Rome, Italy; roberto.disanto@uniroma1.it (R.D.S.); roberta.costi@uniroma1.it (R.C.)
5 Net4Science Academic Spin-Off, Università degli Studi "Magna Græcia" di Catanzaro, Campus "S. Venuta", Viale Europa, 88100 Catanzaro, Italy;
6 Dipartimento di Scienze della Salute, Università degli Studi "Magna Græcia" di Catanzaro, 88100 Catanzaro, Italy; isabella.romeo@unicz.it
7 National Center for Drug Research and Evaluation, Istituto Superiore di Sanità, 00161 Rome, Italy;
marisa.colone@iss.it (M.C.); annarita.stringaro@iss.it (A.S.)
8 Department of Pharmacy and Pharmaceutical Technology and Physical-Chemistry, Faculty of Pharmacy and Food Sciences, University of Barcelona, 27-31 Barcelona, Catalonia, Spain ;
9 Institute of Nanoscience and Nanotechnology (IN[2]UB), 08028 Barcelona, Spain; aespargaro@ub.edu (A.E.); rsabate@ub.edu (R.S.)
* Correspondence: alcaro@unicz.it (SA); luigi.scipione@uniroma1.it (LS).

SUPPORTING INFORMATION

Dixon's plot of compounds 12 and 14 towards *eq*BChE pag. 2
IC_{50} graph of compound 18 towards *eq*BChE pag. 3
***In silico* studies** pag. 4
UV-Vis titration spectra and Job's plots for selected compounds pag. 7

Dixon's plot of compounds 12 and 14 towards *eq*BChE

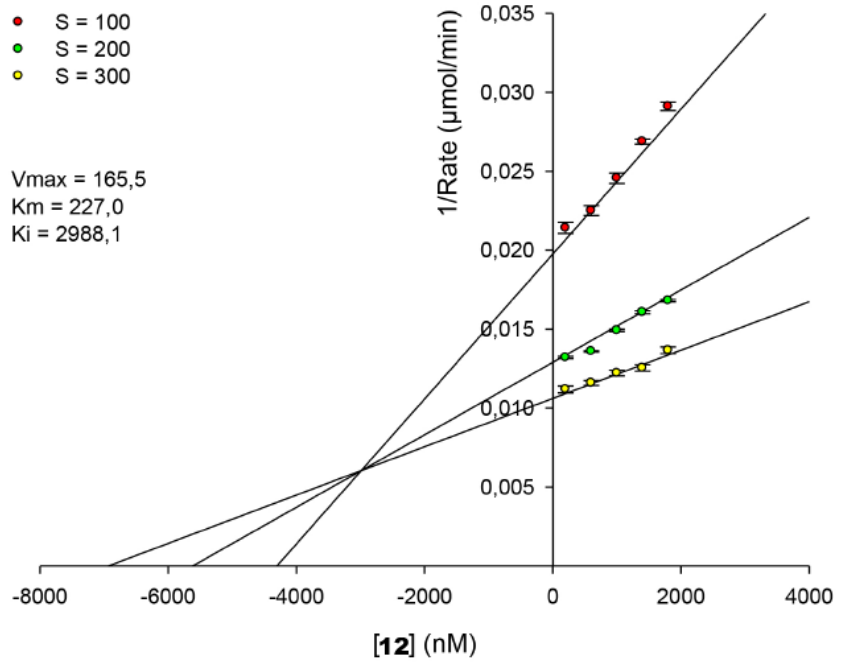

Figure S1. Dixon's plot obtained for **12** (200-1800 nM), in presence of *eq*BChE (0.0833 U/mL) and ASCh (100-300 µM). Competitive inhibition mechanism was observed (Ki = 2.988 ± 0.190 µM, R^2 = 0.987).

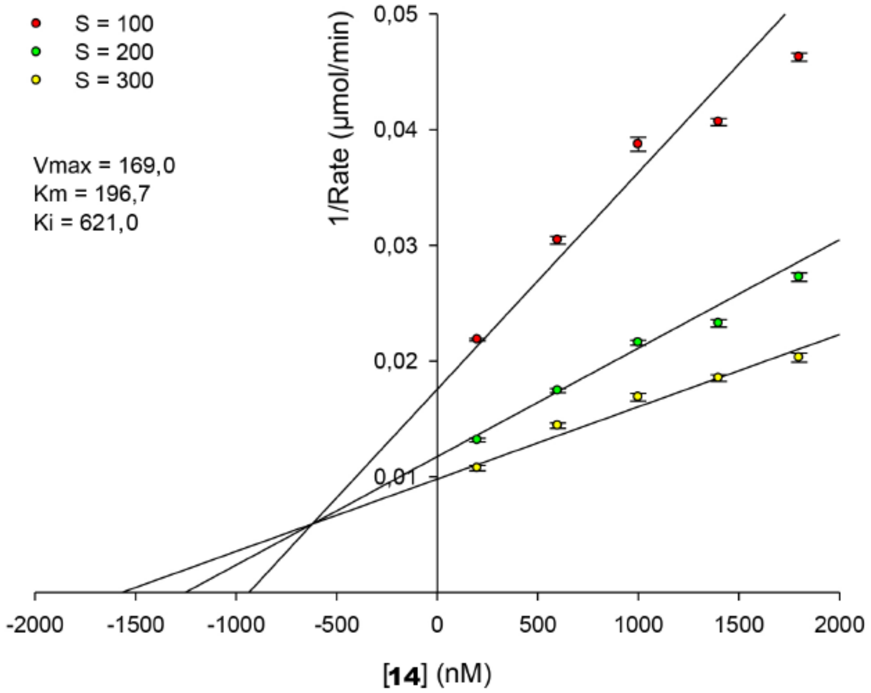

Figure S2. Dixon's plot obtained for **14** (200-1800 nM), in presence of *eq*BChE (0.0833 U/mL) and ASCh (100-300 µM). Competitive inhibition mechanism was observed (Ki = 0.621 ± 0.043 µM, R^2 = 0.980).

IC_{50} graph of compound 18 towards *eq*BChE

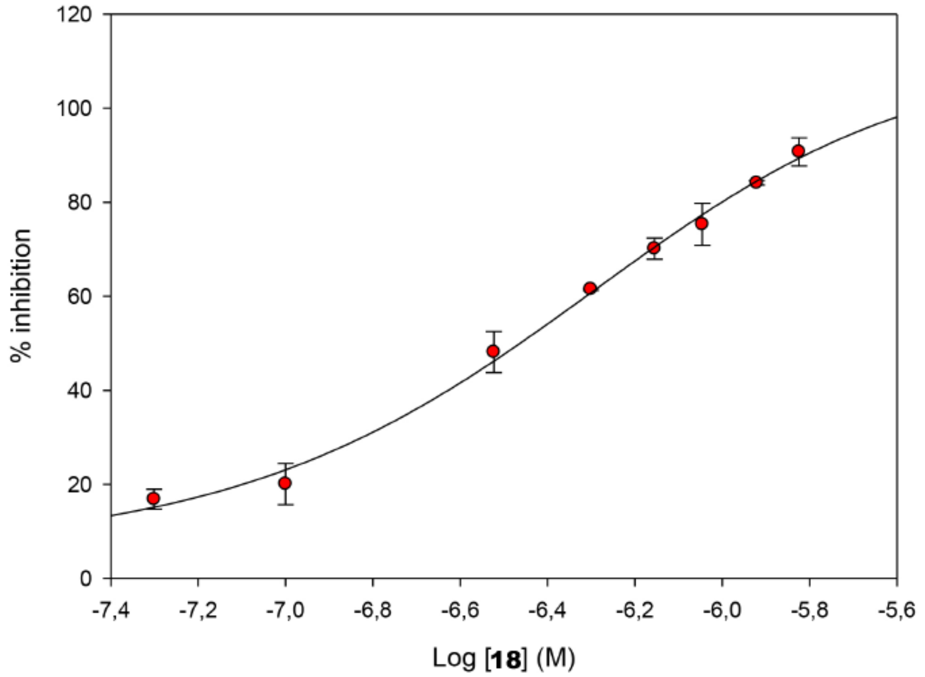

Figure S3. IC_{50} graph for compound **18** (50-1500 nM), in presence of *eq*BChE (0.0833 U/mL) and ASCh (100 µM). The IC_{50} value extrapolated from the graph is 454 ± 82 nM.

In silico studies

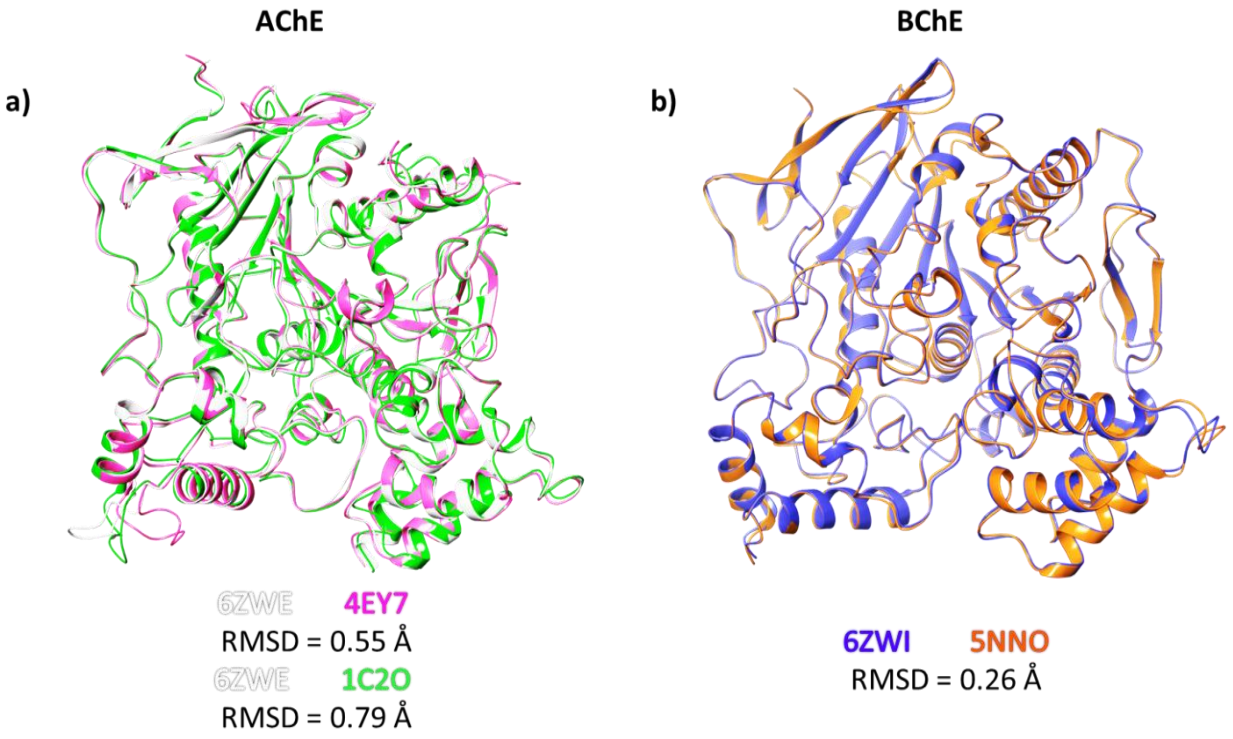

Figure S4. a) Superposition of *Electrophorus electricus* AChE (PDB code: 1C2O) and human AChE (PDB code: 4EY7) with human AChE (PDB code: 6ZWE) and **b)** superposition of human BChE (PDB code: 5NN0) and human BChE (PDB code: 6ZWI) generated by Protein Structure Alignment.

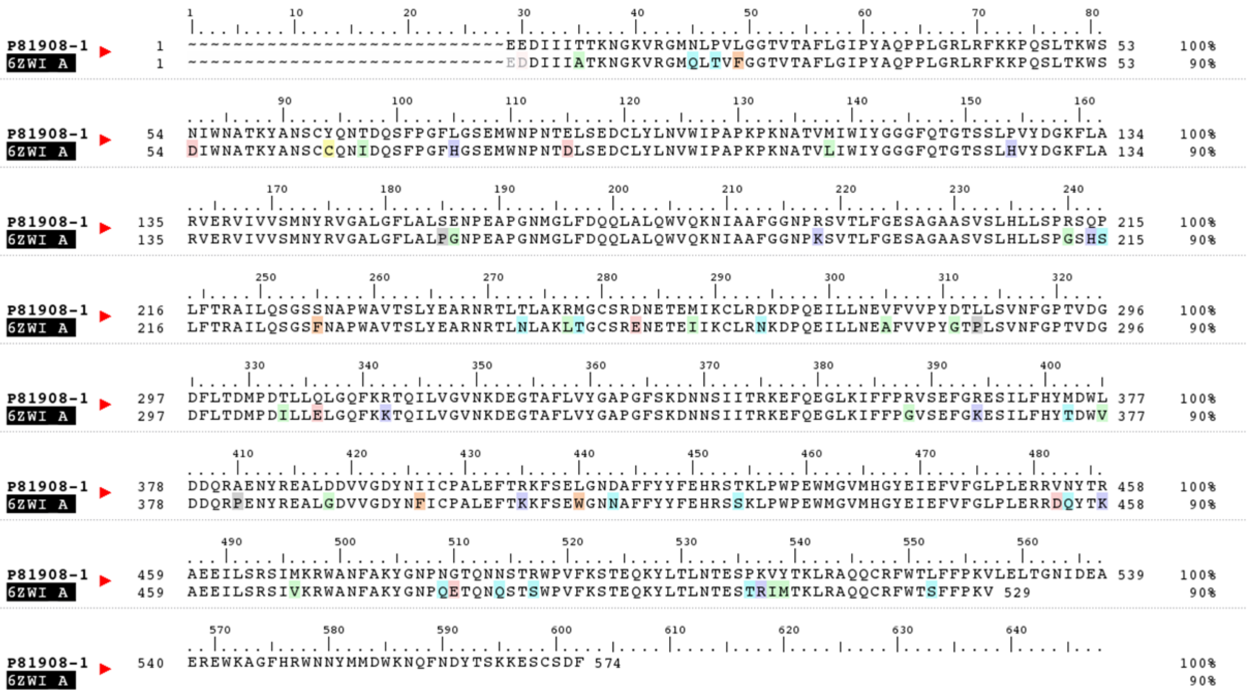

Figure S5. Sequence alignment of equine BChE (entry BuCE_Ecuus, P81908) with human BChE (PDB code: 6ZWI) generated by ClustalW algorithm. Among the sequence, only the different residues are coloured. The percentage identity of the aligned sequence to the selected model is reported.

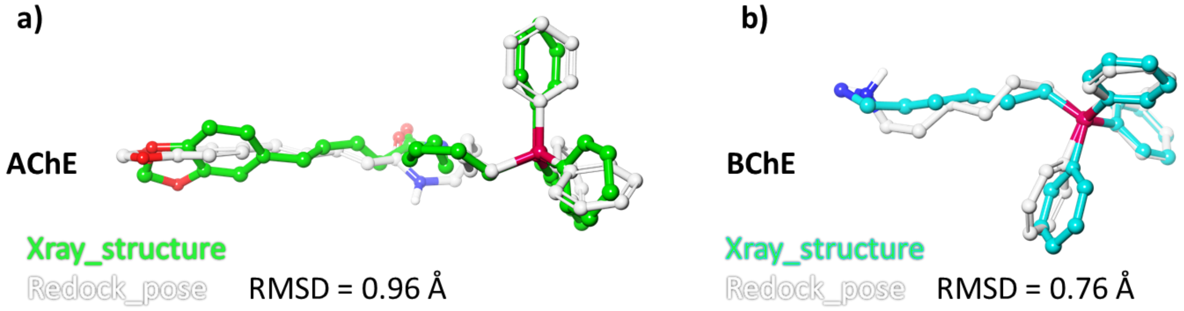

Figure S6. Superimposition of the re-docked pose (gray carbons) and co-crystallized ligand of **a)** *h*AChE and **b)** *h*BChE receptors, represented, respectively, in green and blue carbons. RMSD was found to be 0.96 Å for *h*AChE ligand and 0.76 Å for *h*BChE ligand.

Table S1. GlideScore values (Kcal/mol) of compounds **3-19** for binding *h*AChE and *h*BChE and re-docking values of co-crystallized ligands of 6ZWE and 6ZWI PDB structures.

**Compound**		**GlideScore (Kcal/mol)**	
		***h*AChE**	***h*BChE**
**Pyrimidine amide derivatives**	**3**	-6.37	-6.61
	**4**	-7.87	-7.31
	**5**	-8.02	-7.81
	**6**	-8.04	-7.78
	**7**	-7.68	-7.82
	**8**	-7.87	-7.76
	**9**	-8.07	-7.55
	**10**	-6.18	-5.54
	**11**	-8.77	-8.24
**Pyridine amide derivatives**	**12**	-6.38	-6.68
	**13**	-8.23	-7.67
	**14**	-8.05	-8.13
	**15**	-8.22	-8.46
**Carbamate derivatives**	**16**	-7.31	-7.90
	**17**	-7.31	-7.10
	**18**	-7.59	-8.49
	**19**	-7.65	-7.67
**X-ray ligand**	**6ZWE**	-10.78	-
	**6ZWI**	-	-7.71

Table S2. Molecular Surface Area (Å^3) of the best docking poses of the **3-19** compounds into AChE and BChE binding pocket.

Compound	Molecular Surface Area (Å^3)	
	AChE	BChE
**3**	313.06	320.23
**4**	332.09	341.92
**5**	414.72	429.86
**6**	343.74	354.38
**7**	338.51	342.67
**8**	334.57	343.06
**9**	337.76	348.65
**10**	317.98	319.84
**11**	343.61	356.03
**12**	316.54	335.67
**13**	341.36	346.47
**14**	409.92	436.15
**15**	332.62	359.54
**16**	320.26	333.34
**17**	349.98	356.32
**18**	335.73	336.52
**19**	341.99	359.64
**Average value**	342.56	354.13

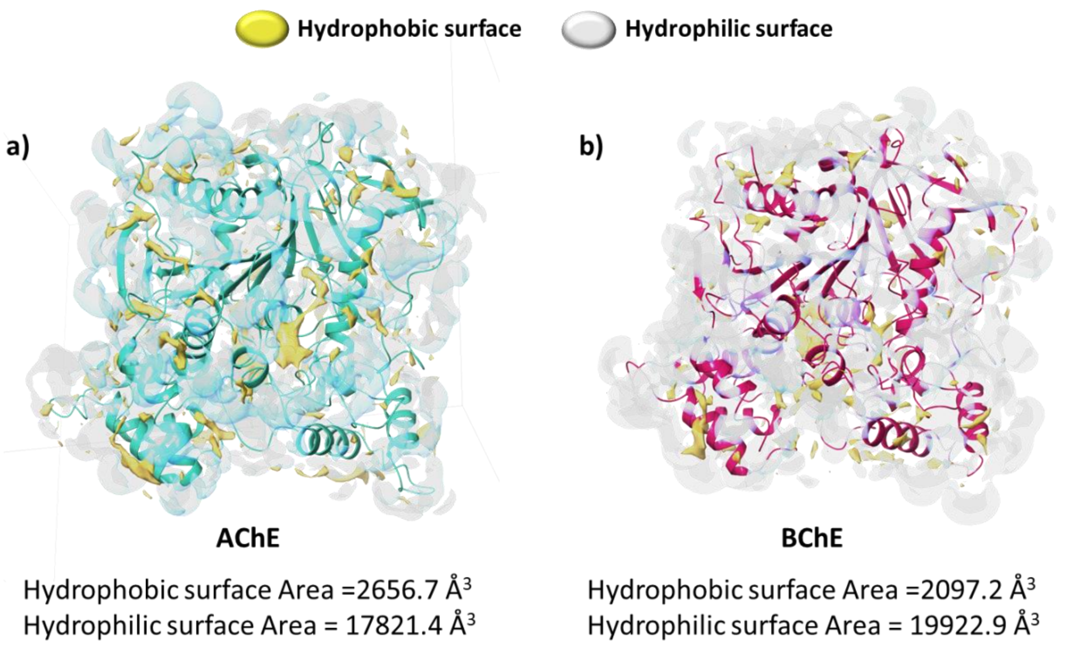

Figure S7. Views of Hydrophobic and Hydrophilic surface area in yellow and grey solid surface, respectively, of **a)** *h*AChE (turquoise cartoon) and **b)** *h*BChE receptors (pink cartoon).

UV-Vis titration spectra and Job's plots for selected compounds

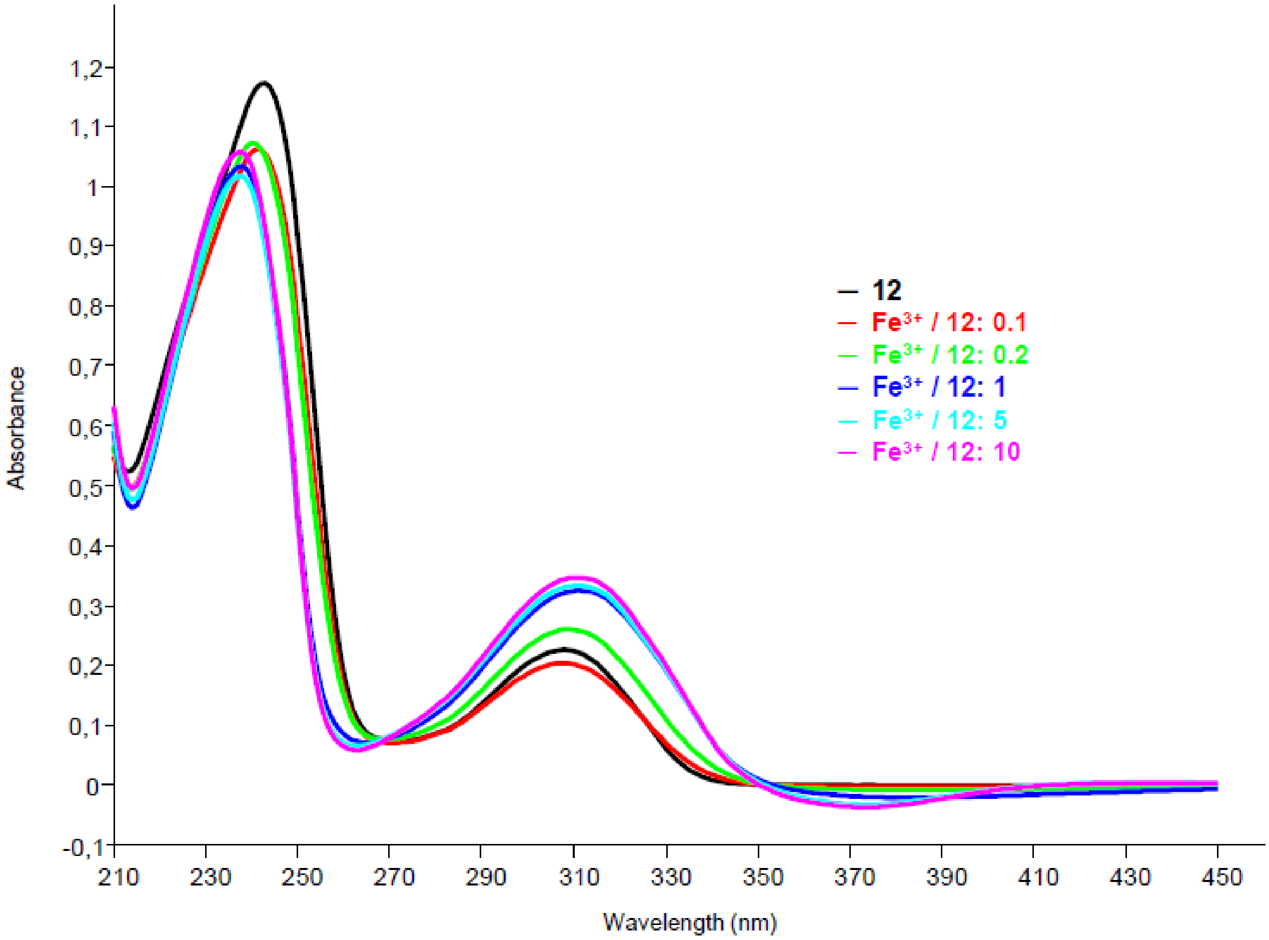

Figure S8. UV-Vis titration of ligand **12** with Fe^{3+}. The greatest variations in spectra with increasing amount of metal are observed at 251 nm, with a reduction of absorbance, and at 320 nm, with an increase of absorbance. There are two isosbestic points at 267 and 350 nm.

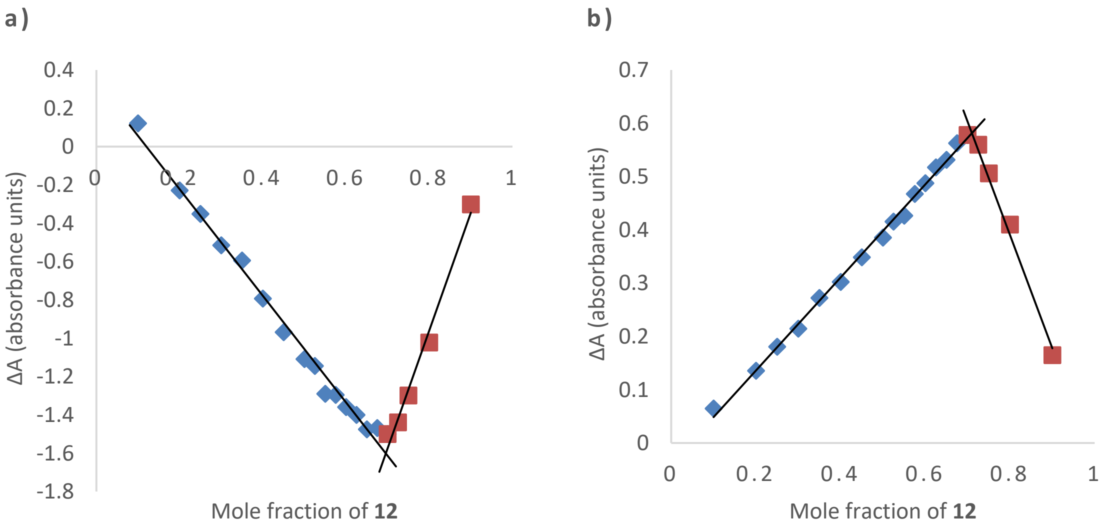

Figure S9. Job's plot of compound **12** in presence of Fe^{3+}: variation of the absorbance (ΔA) at the wavelength of 251 nm in **a)** or at 320 nm in **b)**, in ordinate, versus the mole fraction of **12**, in abscissa. X (mole fraction that causes the maximum variation of absorbance) = 0.69 in **a)**, 0.71 in **b)**; n (number of ligand molecules per cation) = 2.

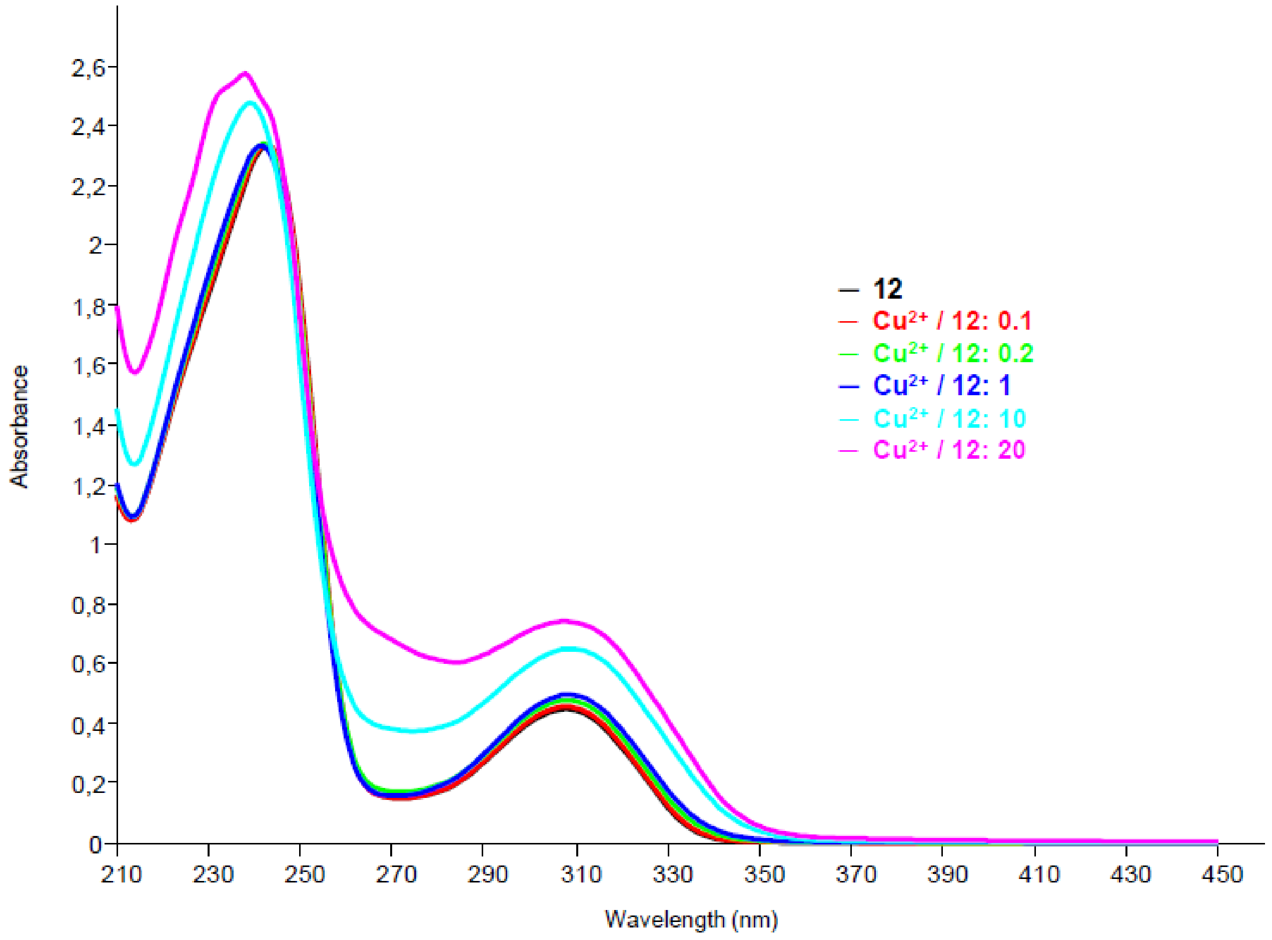

Figure S10. UV-Vis titration of ligand **12** with Cu^{2+}. The greatest variations in spectra with increasing amount of metal are observed at 235, 270 and 310 nm, with an increase of absorbance.

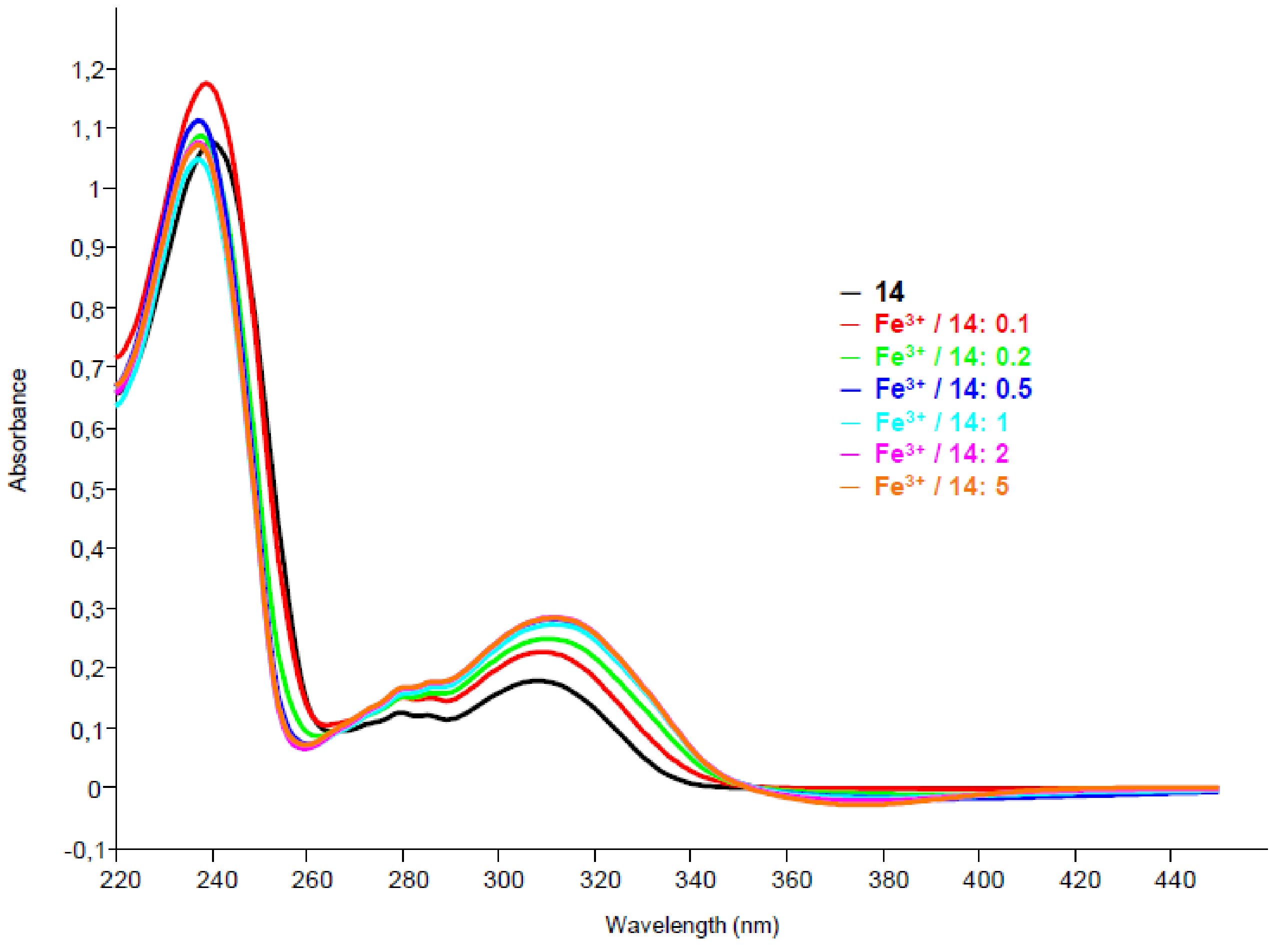

Figure S11. UV-Vis titration of ligand **14** with Fe^{3+}. The greatest variation in spectra with increasing amount of metal is observed at 310 nm, with an increase of absorbance. There are two isosbestic points at 266 and 350 nm.

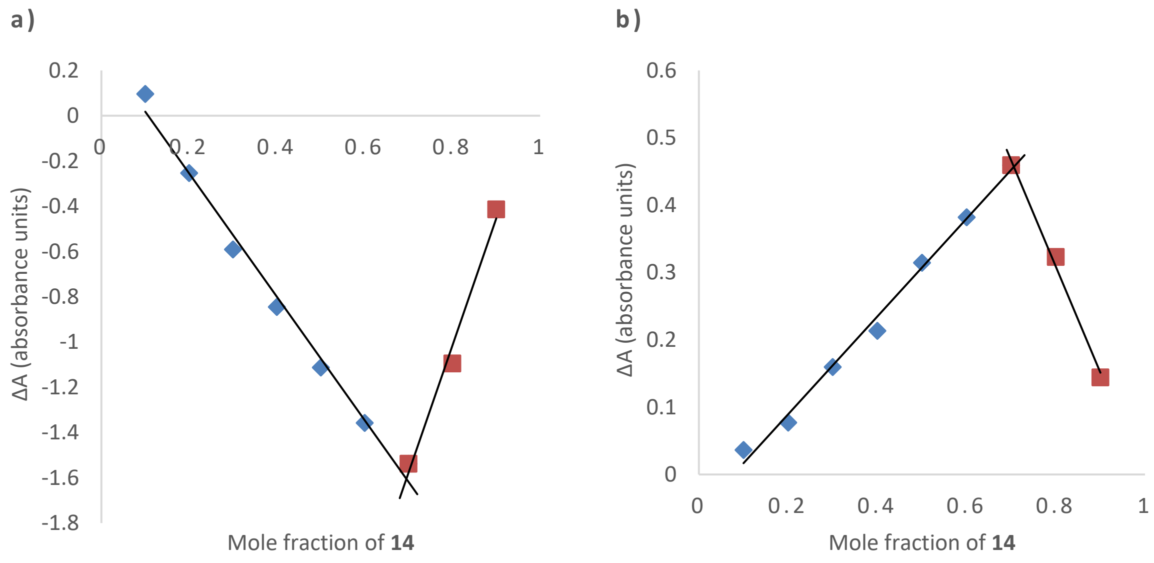

Figure S12. Job's plot of compound **14** in presence of Fe^{3+}: variation of the absorbance (ΔA) at the wavelength of 250 nm in **a)** or at 310 nm in **b)**, in ordinate, versus the mole fraction of **14**, in abscissa. X (mole fraction that causes the maximum variation of absorbance) = 0.70 in **a)**, 0.71 in **b)**; n (number of ligand molecules per cation) = 2.

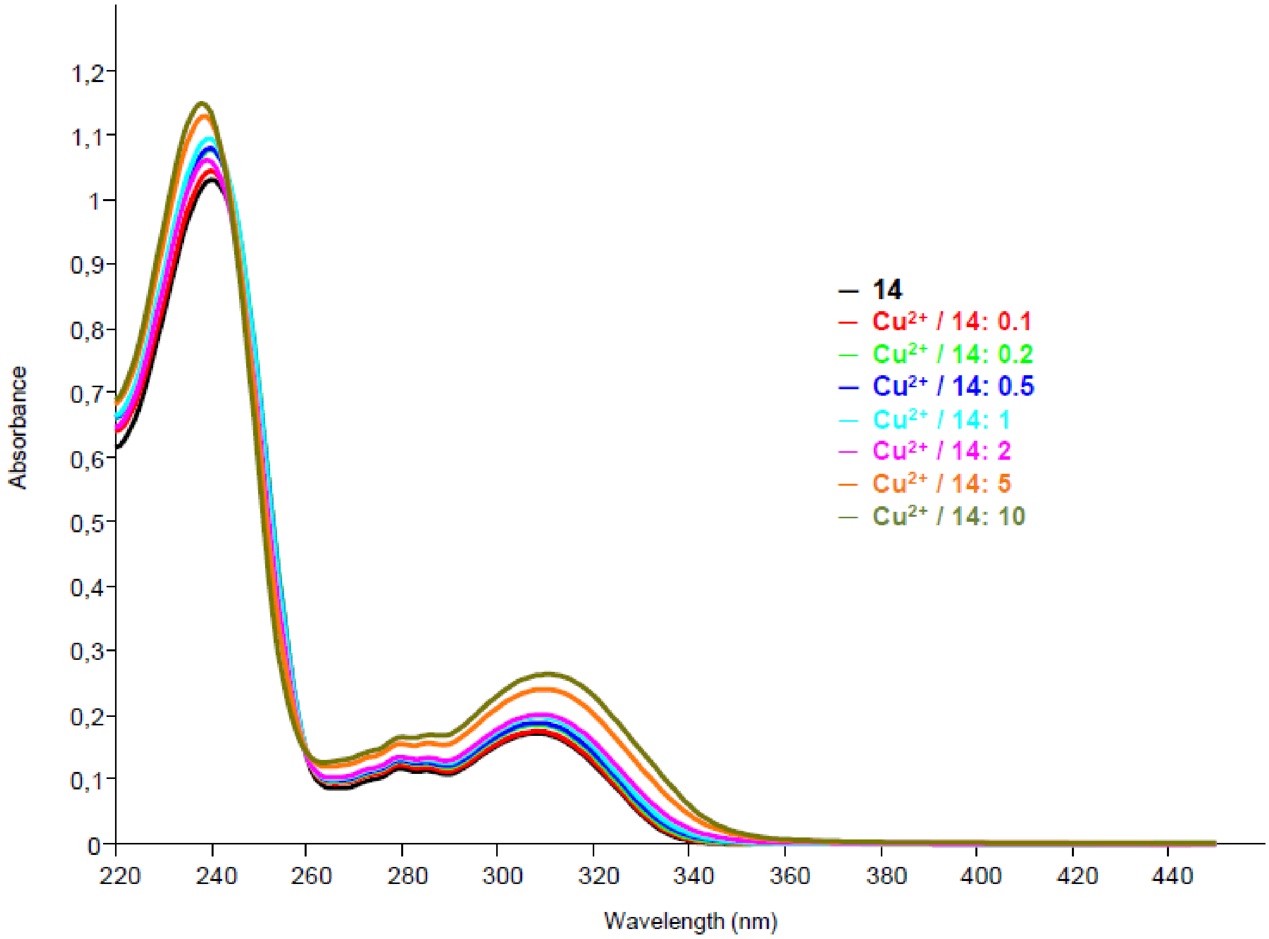

Figure S13. UV-Vis titration of ligand **14** with Cu^{2+}. The greatest variation in spectra with increasing amount of metal is observed at 310 nm, with an increase of absorbance. There is an isosbestic point at 260 nm.

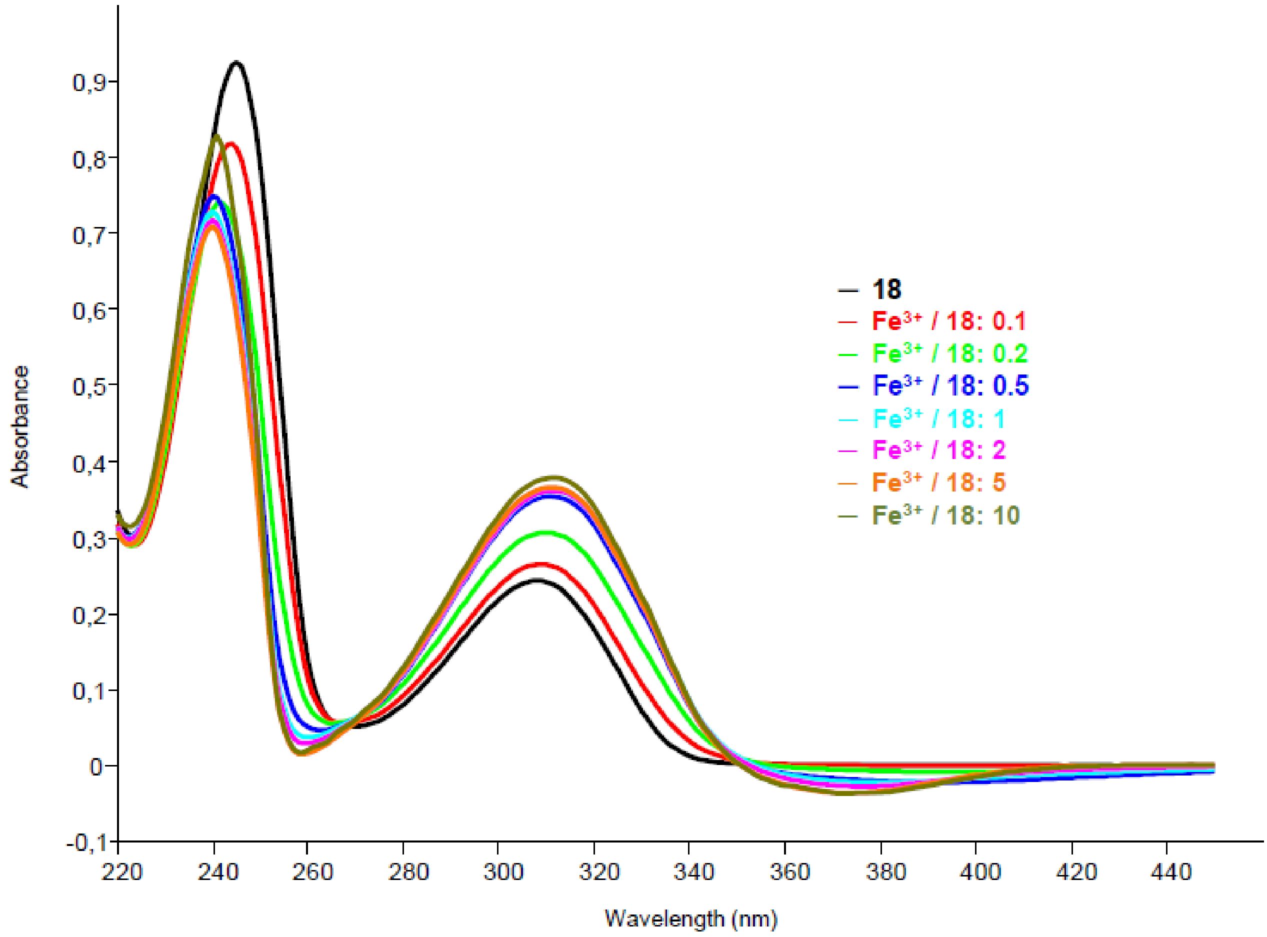

Figure S14. UV-Vis titration of ligand **18** with Fe^{3+}. The greatest variations in spectra with increasing amount of metal are observed at 245 nm, with a reduction of absorbance, and at 310 nm, with an increase of absorbance. There are two isosbestic points at 267 and 353 nm.

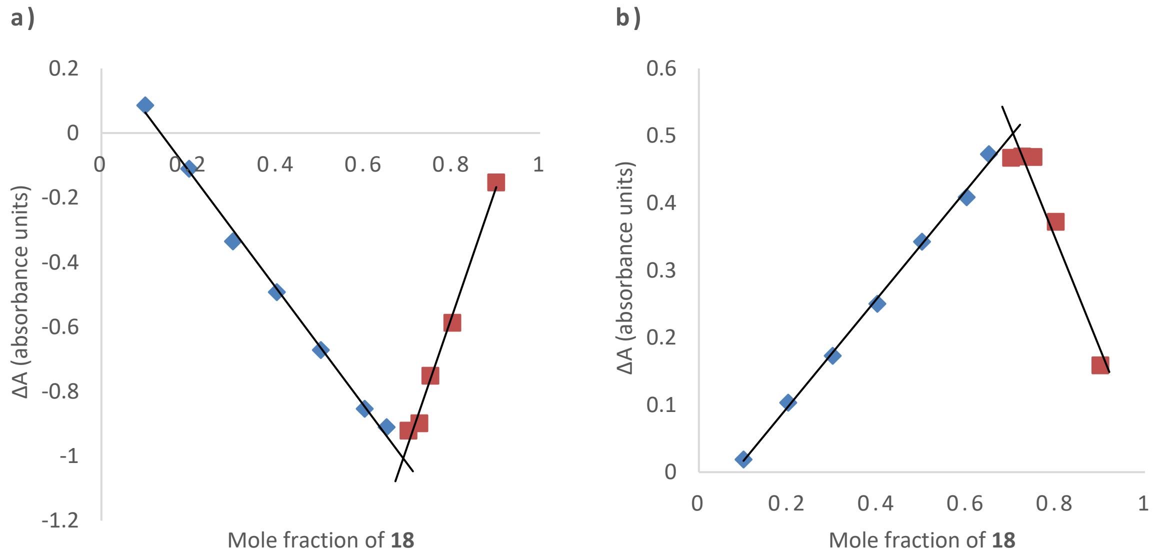

Figure S15. Job's plot of compound **18** in presence of Fe^{3+}: variation of the absorbance (ΔA) at the wavelength of 245 nm in **a)** or at 310 nm in **b)**, in ordinate, versus the mole fraction of **18**, in abscissa. X (mole fraction that causes the maximum variation of absorbance) = 0.69 in **a)**, 0.70 in **b)**; n (number of ligand molecules per cation) = 2.

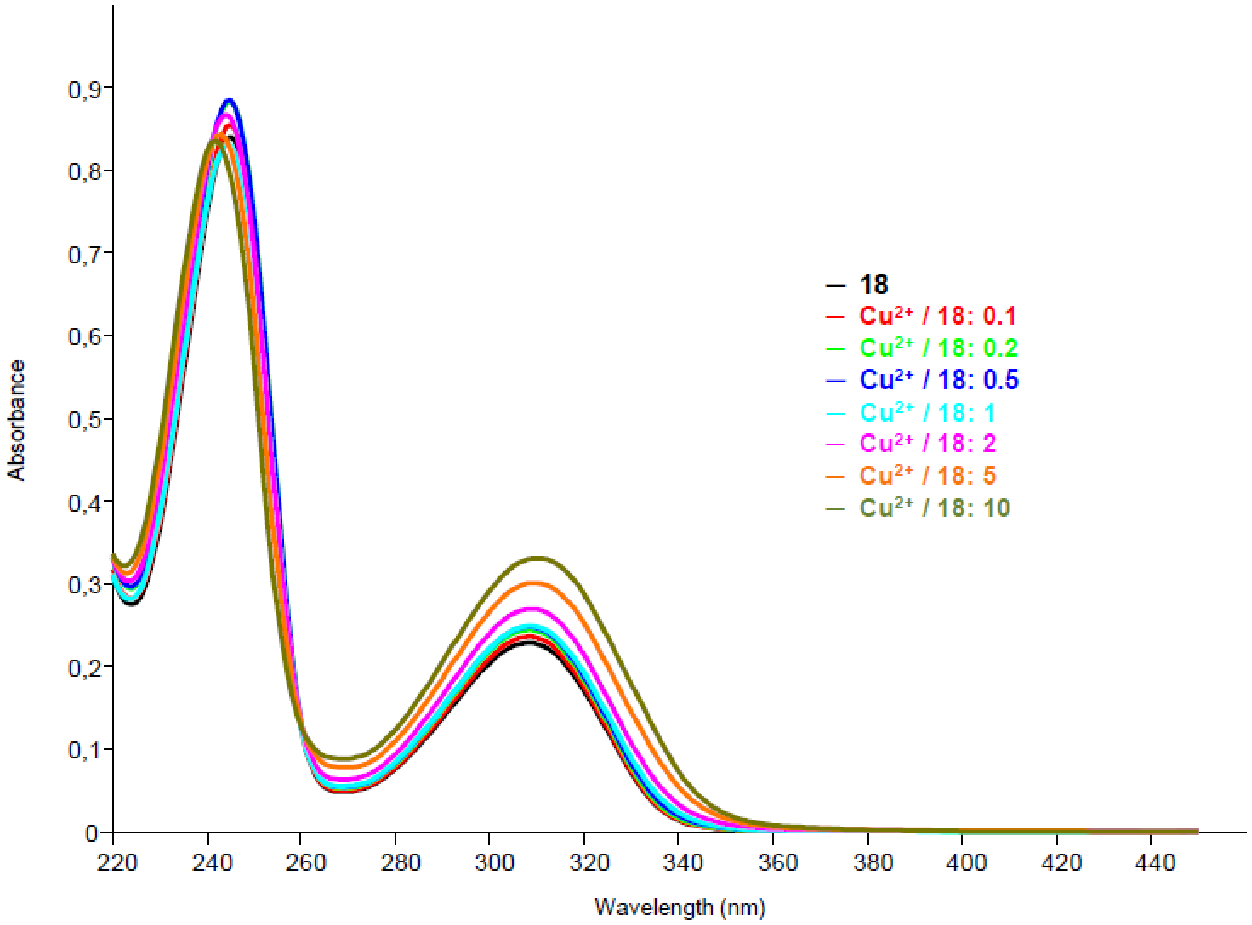

Figure S16. UV-Vis titration of ligand **18** with Cu^{2+}. The greatest variations in spectra with increasing amount of metal are observed at 270 and 310 nm, with an increase of absorbance. There is an isosbestic point at 261 nm.

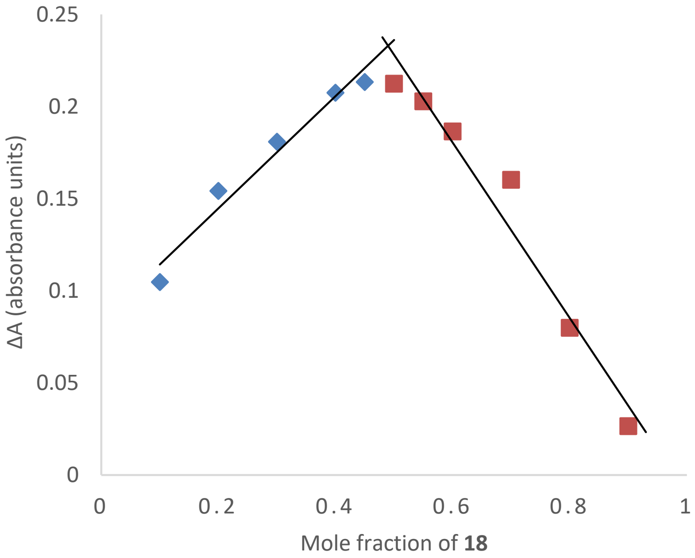

Figure S17. Job's plot of compound **18** in presence of Cu^{2+}: variation of the absorbance (ΔA) at the wavelength of 330 nm, in ordinate, versus the mole fraction of **18**, in abscissa. X (mole fraction that causes the maximum variation of absorbance) = 0.49; n (number of ligand molecules per cation) = 1.
